# Transcriptomic Analysis Revealed Reactive Oxygen Species Scavenging Mechanisms Associated With Ferrous Iron Toxicity in Aromatic Keteki Joha Rice

**DOI:** 10.3389/fpls.2022.798580

**Published:** 2022-02-24

**Authors:** Preetom Regon, Sangita Dey, Mehzabin Rehman, Amit Kumar Pradhan, Umakanta Chowra, Bhaben Tanti, Anupam Das Talukdar, Sanjib Kumar Panda

**Affiliations:** ^1^Department of Life Science and Bioinformatics, Assam University, Silchar, India; ^2^Plant Molecular Biology Laboratory, Department of Botany, Gauhati University, Guwahati, India; ^3^Department of Botany, Pragjyotish College, Guwahati, India; ^4^Department of Botany, Guwahati College, Guwahati, India; ^5^Department of Biochemistry, Central University of Rajasthan, Ajmer, India

**Keywords:** Fe^2+^ toxicity, RNA-Seq, transcriptome, Fe homeostasis, ROS

## Abstract

Lowland acidic soils with water-logged regions are often affected by ferrous iron (Fe^2+^) toxicity, a major yield-limiting factor of rice production. Under severe Fe^2+^ toxicity, reactive oxygen species (ROS) are crucial, although molecular mechanisms and associated ROS homeostasis genes are still unknown. In this study, a comparative RNA-Seq based transcriptome analysis was conducted to understand the Fe^2+^ toxicity tolerance mechanism in aromatic Keteki Joha. About 69 Fe homeostasis related genes and their homologs were identified, where most of the genes were downregulated. Under severe Fe^2+^ toxicity, the biosynthesis of amino acids, RNA degradation, and glutathione metabolism were induced, whereas phenylpropanoid biosynthesis, photosynthesis, and fatty acid elongation were inhibited. The *mitochondrial iron transporter (OsMIT), vacuolar iron transporter 2 (OsVIT2), ferritin (OsFER), vacuolar mugineic acid transporter (OsVMT), phenolic efflux zero1 (OsPEZ1), root meander curling (OsRMC)*, and *nicotianamine synthase (OsNAS3)* were upregulated in different tissues, suggesting the importance of Fe retention and sequestration for detoxification. However, several antioxidants, ROS scavenging genes and abiotic stress-responsive transcription factors indicate ROS homeostasis as one of the most important defense mechanisms under severe Fe^2+^ toxicity. Catalase (CAT), glutathione (GSH), ascorbate peroxidase (APX), monodehydroascorbate reductase (MDHAR), dehydroascorbate reductase (DHAR), and glutathione reductase (GR) were upregulated. Moreover, abiotic stress-responsive transcription factors, no apical meristem (NAC), myeloblastosis (MYB), auxin response factor (ARF), basic helix-loop-helix (bZIP), WRKY, and C2H2-zinc finger protein (C2H2-ZFP) were also upregulated. Accordingly, ROS homeostasis has been proposed as an essential defense mechanism under such conditions. Thus, the current study may enrich the understanding of Fe-homeostasis in rice.

## 1. Introduction

Iron (Fe) is an essential micronutrient, participating in various vital processes in cell metabolism due to its redox status between ferrous (Fe^2+^) and ferric (Fe^3+^) forms. Fe functions as an electron acceptor or donor actively participating in photosynthesis and respiration (Kobayashi and Nishizawa, 2012; Zhai et al., 2014). Lowland and flooded soil are poor in oxygen concentration and become acidic. The flooding of rice fields leads to the reduction of Fe^3+^ to Fe^2+^, which becomes highly toxic to paddy crops. Fe^2+^ toxicity is a major cause of abiotic stress affecting large rice-growing areas, especially in Asian countries (Becker and Asch, 2005). Depending on stress intensities, a loss of 12% to 100% has been reported on rice yields (Sahrawat, 2005; Audebert and Fofana, 2009). Fe^2+^ toxicity is a severe agricultural constrain that generally occurs in acidic soils. Thus, understanding the underlying molecular mechanisms associated with their adaptation may help to build strategies for future rice breeding programs for Fe^2+^ toxicity tolerance.

Angiosperms plants have evolved two acquisition strategies to overcome the limited Fe availability *viz*., strategy I and II. The strategy I is based on the acidification of rhizospheres by releasing protons from the roots, which reduces Fe^3+^ to Fe^2+^ and its transportation through iron-regulated transporter (IRT) across the root plasma membrane. Strategy II is based on the uptake of Fe^3+^ phytosiderophore complexes. Although being a Strategy II plant, rice (*Oryza sativa* L.) possesses Fe^2+^ transporter genes (*OsIRT1* and *OsIRT2*) that can directly uptake Fe^2+^ from the soil (Bughio, 2002; Ishimaru et al., 2006). The mechanism of Fe^2+^ toxicity tolerance in rice plants has been highlighted in several studies (Asch et al., 2005; Deng et al., 2010; Wu et al., 2017; Aung et al., 2018; Aung and Masuda, 2020). Rice plants possess several defense mechanisms to cope with Fe^2+^ toxicity. The defense mechanism of rice plants for Fe^2+^ tolerance can be divided into four mechanisms (Aung and Masuda, 2020). Defense 1 (Fe exclusion from roots) is a root-based tolerance mechanism where Fe plaques (Fe^3+^ precipitation) on the root surface acts as a barrier to the uptake of Fe^2+^ into root tissues. Fe plaque is formed due to the rhizospheric oxidation of Fe^2+^ to Fe^3+^ by oxygen transport from shoots to roots (Asch et al., 2005; Becker and Asch, 2005). Defense 2 (Fe retention in roots and suppression of Fe translocation to shoots) where discrimination center and ferritin (*OsFER*) plays an important role by retaining excess Fe in root and avoiding translocation into the shoot. Defense 3 (Fe compartmentalization in shoots) includes compartmentalization, disposal, or storage of Fe inside shoots by vacuolar iron transporter (*OsVIT1*/*OsVIT2*), and *OsFER1/2*. Defense 4 [reactive oxygen species (ROS) detoxification] includes enzymatic detoxification, scavenging of ROS by antioxidants. Defense 1–3 was highlighted to work on mild to moderate Fe excess conditions and do not seriously affect rice growth. In contrast, defense 4 was hypothesized to work at a molecular level under Fe severe conditions that causes bronzing and inhibit plant growth and development (Aung and Masuda, 2020). ROS antioxidants like glutathione S-transferase (GST) and ascorbate oxidase were reported to play an important role in shoot-based Fe tolerance in rice plants (Wu et al., 2017). Besides, under severe Fe excess conditions, cytochrome P450 family proteins, *OsNAC4, OsNAC5*, and *OsNAC6*, played an important role in alleviating ROS (Aung and Masuda, 2020). The elucidation of the genes involved in response to Fe-toxicity is fundamental to understanding the mechanism that confers tolerance to stress and the development of tolerant cultivars. With the advancement of next generation sequencing (NGS) technology, the RNA-seq technique has become a valuable tool for transcriptional profile analysis, providing a better understanding of gene responses to Fe^2+^ toxicity. To date, many studies on the transcriptional profile of rice under Fe-stress have been done. However, there is still a broad spectrum that needs better clarification to understand the Fe stress mechanism, which can help to understand the unsolved queries related to Fe toxicity in rice.

In this study, the transcriptome of aromatic rice was analyzed in both root and leaves using RNA-Seq. Along with the common Fe-homeostasis-related genes, the study identified several differentially expressed genes (DEGs) related to the ROS scavenging system. The results provided a comprehensive transcriptome resource that may enrich the understanding of Fe-homeostasis under Fe^2+^ toxicity conditions.

## 2. Materials and Methods

### 2.1. Plant Growth and Treatment

The Keteki Joha rice variety seeds were collected from Regional Agricultural Research Station, Assam Agricultural University, Titabor, Assam, India (26.575626, 94.183318 and 99 m). Seeds were surface sterilized with 0.01% sodium hypochlorite (NaOCI) and germinated in Petri dishes by incubation at 28°C for 3-5 days. Germinated seedlings were grown in a plastic pots, containing 400 ml of Hoagland nutrient solutions, prepared in distilled water (Hoagland and Snyder, 1933). Rice seedlings were grown under controlled growth chamber Jeiotech GC-300TLH with 14 h light (Temp 28°C, RH 70%, illumination 8000 lux) and 10 h dark (Temp 22°C, RH 60%), conducted at Assam University Silchar, India (24.686545, 92.751699 and 31 m). About 3 weeks old rice seedlings were treated with 2.5 mM of FeEDTA for 72 h at pH 4.5. The FeEDTA solution was prepared as described by Steiner and van Winden (1970) with few modifications. For the mature stage, rice plants were grown in a pot [20.5 (D) X 15 CM (Height)] containing approximately 3.5 kg (dry weight) of alluvial soil collected from Silcoorie Grant, Silchar, India (24.728505, 92.747980 and 21 m). About 60–70-day old plants were treated with 5 L of 2.5 mM FeEDTA at pH 4.5 (prepared with Hoagland) for 2 weeks, and leave samples were harvested for RNA isolation and sequencing (Supplementary Figure S1). The FeEDTA solution was changed every 96 h. Three utterly independent pot experiments were considered as biological replicates (Supplementary Figure S2).

### 2.2. RNA Sequencing

RNA was isolated from both roots and leaves with 3 biological replicates using the RNAqueous Phenol-free total RNA isolation kit of Invitrogen, Thermo Fisher Scientific. RNA Integrity Number (RIN) value of more than 6.5 was used to prepare the sequencing library prepared by Truseq Stranded RNA Library Prep Kit-Plant (Illumina, USA). Finally, 100–150 bp RNA Seq was performed by Illumina HiSeq 2000 platform (Illumina, USA).

### 2.3. Data-processing, Assembly, and Differential Expression

Basic statistics of the sequencing raw reads were accessed by FastQC v 0.11.9 (FastQC, 2010). Raw reads were trimmed with Trimmomatic v 0.39 to remove sequencing adapters (Bolger et al., 2014). Reference-based assembly was performed by STAR v 2.7.1a, where Samtools v 1.7 was used to manipulate the bam files (Li et al., 2009; Dobin et al., 2013). Rice Genome Annotation Project was used as a reference sequence database for assembly (Kawahara et al., 2013). Transcript abundance was quantified using the featureCounts v 2.0.0 program of the Subread package (Liao et al., 2014). Differential gene expression was performed using the DESeq2 v 1.32.0 package of the R program (Love et al., 2014; R Core Team, 2021). Alternative shrinkage was estimated by apeglm method (Zhu et al., 2018). Adjusted *p*-value of ≤ 0.05 was considered as a significant difference in expression between groups. EnhancedVolcano v 1.10.0 and ComplexHeatmap v 2.8.0 package of R was used to represent the volcano and heatmap plots of the DEGs (Gu et al., 2016; Blighe et al., 2021).

### 2.4. Annotation

Transcripts were annotated using an in-house pipeline. Briefly, rice annotation data available in the UniProt database were downloaded, and local BLASTX was performed (Altschul et al., 1990; Apweiler et al., 2009). The best scoring results were merged with the transcript using the R program (R Core Team, 2021). Gene ontology (GO), sub-cellular localization, cross reference KEGG ID, and Pfam were considered during the UniProt annotation. Additional gene description was assigned using the gene annotation data available in the Rice Genome Annotation Project (Kawahara et al., 2013). Also, the corresponding RAPD gene id and gene symbol were assigned to the annotated transcript by using the R program (Sakai et al., 2013).

### 2.5. Gene Set Enrichment Analysis

Gene set enrichment analysis was done with the topGO of the R package (Alexa and Rahnenführer, 2020). The GO term of each locus was obtained from Rice Genome Annotation Project, and molecular function, biological process, and cellular component were studied for the selected top 1,000 DEGs (500 Up/500 down). GO terms were ranked by employing the Fishers' exact statistical test and classic algorithm of topGO. The ggplot2 package of the R program was used to represent the graphical representation (Wickham, 2016).

Metabolic pathways were studied using the KEGG database (Kanehisa and Goto, 2000). Briefly, all the protein sequences of the DEGs were downloaded from the Rice Genome Annotation Project database and re-annotated using GhostKOALA in the KEGG database (Kanehisa et al., 2016). Cross-reference KEGG gene ID of *Oryza sativa, Japonica (osa)*, KEGG entry no T01015, and GHOSTX score ≥100 were considered for pathway mapping. Finally, the KEGG pathway was analyzed using the KEGG mapper. Expression value (log2FoldChange) of the DEGs were integrated into their corresponding KEGG gene of each sample, and multiple states pathway maps were rendered by the Pathview web tool (Luo et al., 2017).

### 2.6. Differential Exon-Usage of the DEGs

Differential exon usage of the DEGs was inferred by Bioconductor package DEXSeq (Anders et al., 2012). Briefly, the mapping was done with STAR, and transcript abundance was quantified with featureCounts as described above. The Subread to DEXSeq python script (https://github.com/vivekbhr/Subread_to_DEXSeq) was used to manipulate the featureCounts data to create the DEXSeq object.

### 2.7. Quantitative Real-Time PCR Analysis

Quantitative real-time PCR was performed to confirm the expression of RNA-Seq. Specific gene expression in both roots and leaves of treated (2.5 mM Fe) was compared with control (70 μM Fe). Briefly, about 100 mg of fresh tissues with 3 biological replicates were grounded to powder with Liquid Nitrogen (LN2). Total RNA was isolated using the RNAqueous Phenol-free total RNA isolation kit of Invitrogen, Thermo Fisher Scientific. The cDNA synthesis was performed using the iScript Reverse Transcription Supermix for RT-qPCR kit, Bio-Rad. Gene-specific primers were designed with Primer3web and a few more were also obtained from research articles (Aung et al., 2018; Che et al., 2019; Nozoye et al., 2019) (Table 1). Two individual actin gene was considered as reference genes for qRT-PCR analysis. The qRT-PCR was performed using the PowerUp SYBR Green Master Mix, Applied Biosystems and performed in QuantStudio 5 Real-Time PCR System, Applied Biosystems. Gene expression was calculated using the comparative CT method and normalized with two reference genes (Taylor et al., 2019).

**Table 1:** List of primers used for the qRT-PCR.

**Gene name**	**Primer sequences (5'–3')**	**References**
OsTom1	Fw- GCCCAAGAACGCCAAAATGA	
	Rv- GGCTTGAAGGTCAACGCAAG	
OsNAS1	Fw- GTCTAACAGCCGGACGATCGAAAGG	
	Rv- TTTCTCACTGTCATACACAGATGGC	
OsB12D	Fw- CCGCCATGACCTTCGTCAC	
	Rv- TGGTACTTCTCACCCTCCTC	
OsVMT	Fw- GGACGATGGGTACTGCATTGAG	Che et al., 2019
	Rv- GGGAACAATGATGATATTGGTAAA	
OsGST44	Fw- GGAGGGAGAGAAGAAGAGCG	
	Rv- TTGGAGGAGAGGAGCAGGG	
OsFER1	Fw- GTGAAGGGCAGTAGTAGGTTTCG	Aung et al., 2018
	Rv- CGCGCGACATACACATGATTCTG	
OsIRT1	Fw- ACCAGATGTTCGAGGGGATG	
	Rv- CTGTTGTCCCTGTACACCCT	
OsYSL15	Fw- AGACGGGACATCTCACCTTG	Che et al., 2019
	Rv- GCCATGTTGCGGTAGATCAG	
OsActin1	Fw- ACACCGGTGTCATGGTCGG	Nozoye et al., 2019
	Rv- ACACGGAGCTCGTTGTAGAA	
OsActin2	Fw- CTTCTAATTCTTCGGACCCAAG	Nozoye et al., 2019
	Rv- CTGGTACCCTCATCAGGCAT	

### 2.8. Statistical Analysis

The statistical analysis of gene expression by qRT-PCR was analyzed with a minimum of 3 biological replicates. The significant mean difference between the treated (2.5 mM Fe) and control (70 μM Fe) was estimated by the student's *t*-test performed in the R program (R Core Team, 2021). Significant differences were represented with asterisks symbol (*p < 0.05; **p < 0.01; ***p < 0.001) compared with control.

## 3. Results

### 3.1. RNA Sequencing and Reference-Based Transcriptome Assembly

**Figure 1:**
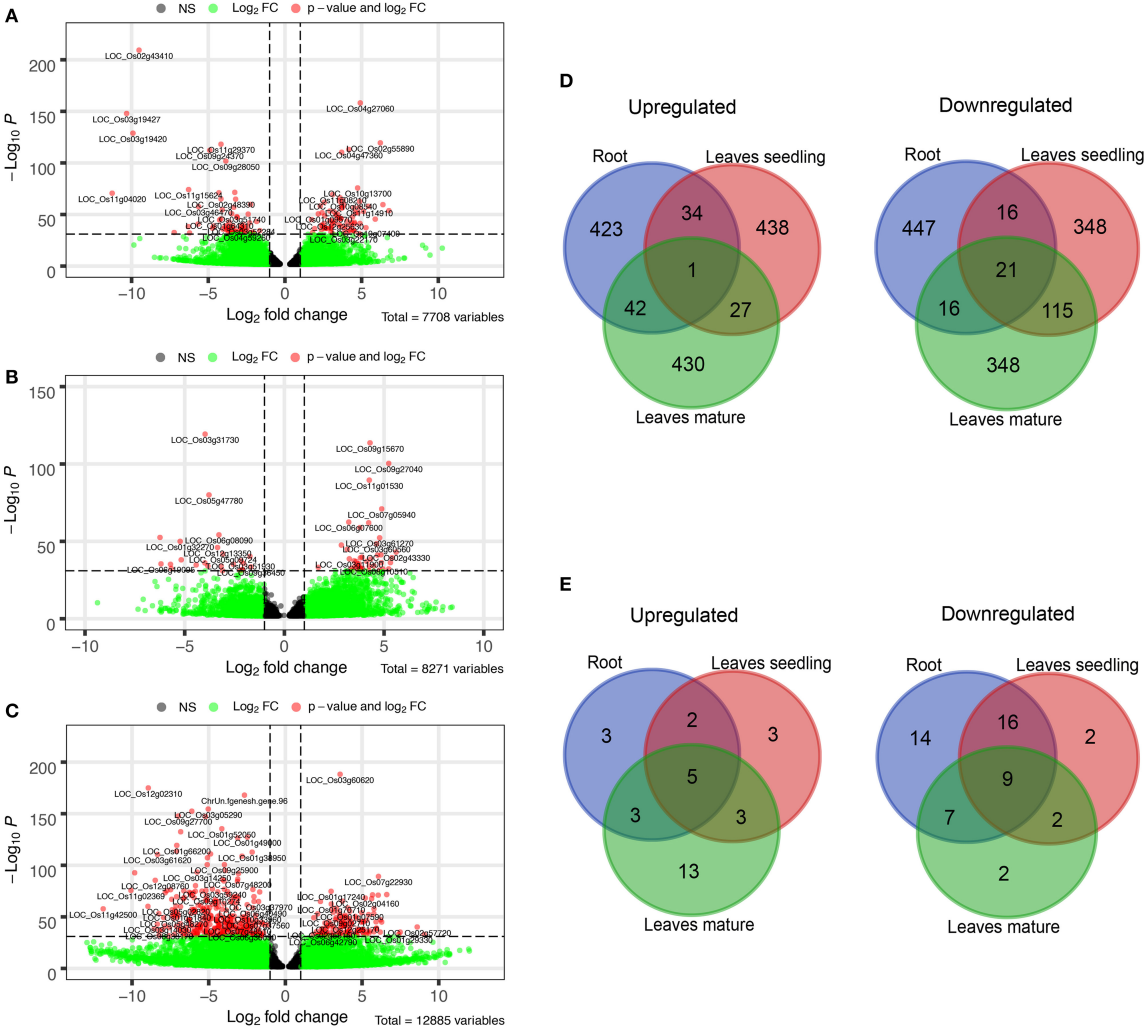
Differentially expressed genes (DEGs) under Fe^2+^ toxicity. **(A–C)** Volcano plots showing the expression of DEGs under different tissue samples. The *p*-value less than 10e-32 were tagged with their gene ID, **(D)** Venn diagram showing the top 500 Up and downregulated common DEGs among the tissue samples, **(E)** Venn diagram of common Fe-homeostasis-related DEGs, expressed in different tissues samples. The Venn diagrams were created by using the Venn diagram software of VIB/UGent Bioinformatics & Evolutionary Genomics.

Growth of the rice plant was significantly inhibited under severe Fe^2+^ toxicity (Supplementary Figure S1). RNA-Seq transcriptome was performed and approximately 9.36–37.40 million 100-150 bp sequence reads were obtained from each sequencing run from which 41.59 to 72.27% were uniquely mapped into the *Japonica* rice genome (Supplementary Tables S1, S2). About 44% of GC content was observed among the sequencing reads. About 7,708 DEGs in roots, 8,271 in leaves seedling, and 12,885 in leaves mature stage were identified (Figures 1A–C).

### 3.2. Differentially Expressed Genes in Roots

**Figure 2:**
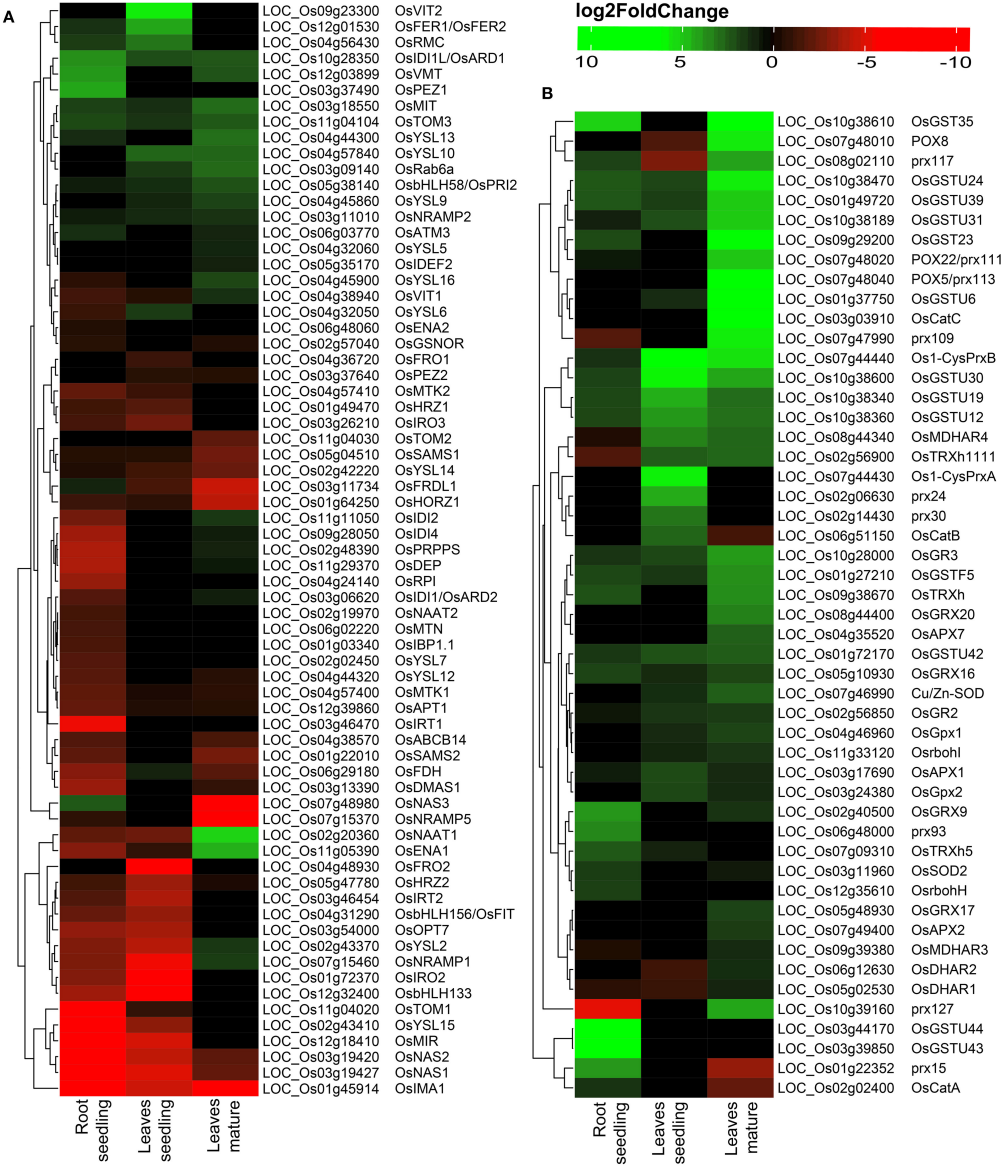
Heatmap of DEGs under Fe^2+^ toxicity. **(A)** Heatmap of Fe-homeostasis-related genes. **(B)** Heatmap of antioxidant and reactive oxygen species (ROS) scavenging related genes. The color scale bar represents the log2FoldChange value of the DEGs.

Among DEGs, a total of 69 Fe homeostasis-related genes and their homologs were identified (Figure 2A). Most of the genes associated with Fe homeostasis were downregulated under Fe^2+^ toxicity. However, 13 Fe homeostasis related genes were upregulated in the roots, of which the genes *phenolic efflux zero1 (OsPEZ1), vacuolar mugineic acid transporter (OsVMT)*, and *acireductone dioxygenase 1/submergence-induced protein 2 (OsARD1/OsSIP2)* were highly upregulated. Other upregulated genes include *nicotianamine synthase 3 (OsNAS3), transporter of mugineic acid 3 (OsTOM3), mitochondrial iron transporter (OsMIT), root meander curling (OsRMC), OsFER1/OsFER2, ABC transporter of mitochondrion 3 (OsATM3), yellow stripe-like 13 (OsYSL13), FRD3-Like Protein 1 (OsFRDL1), natural resistance-associated macrophage protein 2 (OsNRAMP2)*, and *positive regulator of iron homeostasis 2 (OsbHLH58/OsPRI2)*. The Fe^2+^ transporter *OsIRT1, OsIRT2, OsNRAMP1*, and Fe/Mn/Cd transporter *OsNRAMP5* were downregulated. In addition, Fe-phytosiderophore transporters like *OsYSL15* and *OsYSL2* were also downregulated. Other downregulated genes include *OsTOM1, OsNAS1, OsNAS2, mitochondrial iron-regulated gene (OsMIR), iron man (OsIMA1), phosphoribosyl pyrophosphate synthetase (OsPRPPS), dehydratase-enolase-phosphatase (OsDEP), Fe-deficiency-induced protein 4 (OsIDI4), basic helix-loop-helix protein 133 (OsbHLH133), deoxymugineic acid synthase 1 (OsDMAS1), ribose 5-phosphate isomerase (OsRPI), oligopeptide transporter 7 (OsOPT7), formate dehydrogenase (OsFDH), efflux transporter of nicotianamine 1 (OsENA1), iron-related transcription factor 2 (OsIRO2), OsNRAMP1, OsIDI2, FER-like Fe deficiency-induced transcription factor (OsbHLH156/OsFIT)* etc. (Figure 2A). Besides, few Fe homeostasis related genes like *OsVIT2, OsYSL10, small GTP-binding protein (OsRab6a), OsYSL9, OsTOM2, IDE-binding factor 2 (OsIDEF2), OsYSL5, OsPEZ2, ferric reductase oxidase 1 (OsFRO1)*, and *OsFRO2* were not expressed in roots (Figure 2A).

### 3.3. Differentially Expressed Genes in Leaves

Variation was observed among the DEGs in the leaves of seedlings with that of the mature stage. The *OsVIT2* and *OsFER1/OsFER2* were highly upregulated in the seedling stage, whereas they were not differentially expressed in the mature stage (Figure 2A). Other upregulated Fe homeostasis related genes in seedlings include the *OsRMC, OsYSL10, OsIDI1L/OsARD1, OsYSL6, OsRab6a, OsTOM3, OsMIT, OsbHLH58/OsPRI2, OsNRAMP2, OsYSL9*, and *OsFDH*. In contrast, downregulated genes include *OsFRO2, OsIRO2, OsbHLH133, OsNRAMP1, OsNAS1, OsMIR, OsIMA1, OsNAS2, OsYSL2, OsIRT2* etc. (Figure 2A). Among the 69 identified Fe homeostasis-related genes, 27 genes were not expressed in leaves' seedlings (Figure 2A).

In the mature stage, genes like *nicotianamine aminotransferase 1 (OsNAAT1), OsENA1* were highly upregulated, followed by *OsYSL13, OsMIT, OsRab6a, OsYSL10, OsTOM3, OsIDI1L/OsARD1, OsVMT, OsbHLH58/OsPRI2*, etc. (Figure 2A). Most downregulated genes include *OsNAS3, OsIMA1, OsNRAMP5, OsFRDL1, haemerythrin domain containing protein without RING- and Zn-finger 1 (OsHORZ1)*, etc. (Figure 2A). A total of 25 identified Fe homeostasis-related genes were differentially expressed in leaves during the mature stage (Figure 2A).

Comparative analysis of the top 500 up/down regulated genes indicates that genes are distinctly expressed in both roots and leaves. Only one gene was found to be commonly upregulated in the roots and leaves of both seedlings as well as of the mature stage. Similarly, only 21 genes were found to be downregulated in all of the tissue samples (Figure 1D). On comparing the genes associated with Fe homeostasis, it was observed that only 5 genes were commonly upregulated in roots and leaves of seedling and the mature stage. Similarly, 9 genes were expressed as downregulated genes (Figure 1E).

### 3.4. DEGs Involved in ROS and Scavenging

**Figure 3:**
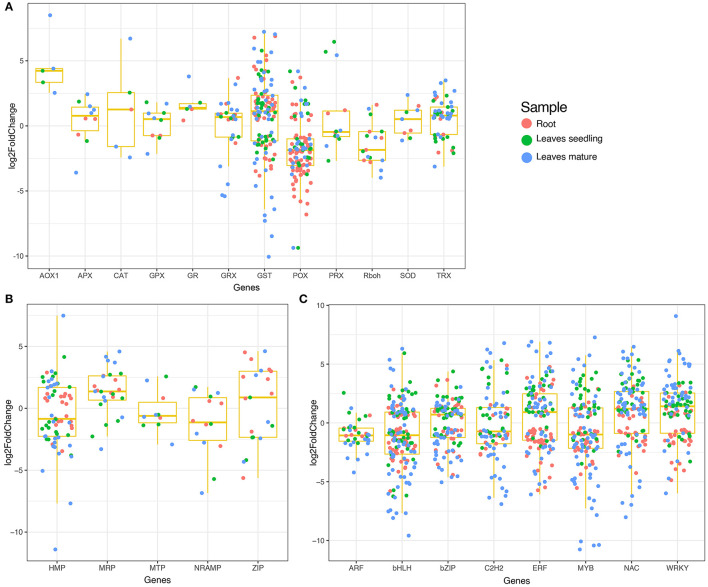
Boxplot showing the expression of DEGs in different tissue samples. **(A)** Expression of antioxidant and ROS scavenging related DEGs, **(B)** Expression of heavy metal related DEGs, and **(C)** Expression of transcription factors.

Reactive oxygen species homeostasis greatly depends on the balance of ROS production and their scavenging. Under Fe^2+^ toxicity, stress-responsive, and source of H_2_O_2_ generation genes such as respiratory burst oxidase homolog (Rboh) were differentially expressed in both roots and leaves (Figure 3A). Only *OsRbohH* was upregulated in roots, whereas *OsRbohF* in leaves of both growth stages (Figure 2B and Supplementary Table S3). Simultaneously, ROS scavenging enzymes such as superoxide dismutase (SOD), catalase (CAT), ascorbate peroxidase (APX), guaiacol peroxidase (GPX), glutathione reductase (GR), peroxiredoxins (PRXs), glutaredoxin (GRX), and peroxidase (POX) were differentially expressed (Figure 3A). The expression of CAT was tissue and growth-specific (Figure 2B and Supplementary Table S3). Only *OsCATA* was upregulated in roots, whereas *OsCatB* and *OsCATC* were upregulated in the leaves seedling and mature stage. Similarly, *OsSOD2* was upregulated in roots, whereas *OsSOD3* was upregulated in leaves of both growth stages (Figure 2B and Supplementary Table S3). Interestingly, *OsGR2* and *OsGR3* were commonly upregulated in all tissue conditions (Figure 2B and Supplementary Table S3). Among various APX, *OsAPX1* and *OsAPX7* were upregulated in all tissue types. In roots, GPX were downregulated, whereas *OsGPX1, OsGPX2*, and *OsGPX3* were upregulated in leaves of both stages. *OsPRXB* was highly upregulated in all tissue samples, whereas *OsPRXA* was specific in the leaves of seedlings. POX and non-enzymatic antioxidant GSTs are comprised of a large gene family and are one of the highest DEGs in this study. Approximately 104 POX and 72 GST genes were differentially expressed across all tissue types (Figure 2B and Supplementary Table S3). *OsPRX15* and *OsPRX93* were highly upregulated in roots, whereas *OsPRX24* and *OsPRX30* in leaves, seedling, and *OsPRX113, OsPRX109, OsPRX110, OsPRX111*, etc., were highly upregulated in the mature stage (Figure 2B and Supplementary Table S3). In roots, *OsGSTU44, OsGSTU43, OsGSTU47, OsGSTU8*, etc., were highly upregulated. Similarly, *OsGSTU30, OsGSTU19, OsGSTU36, OsGSTU12*, etc., were upregulated in leaves seedlings, whereas *OsGSTU8, OsGSTU6, OsGSTU17, OsGSTU24*, etc. were upregulated in the mature stage (Figure 2B and Supplementary Table S3). These results suggested that the enzymatic pathways GST, POX genes play a crucial role in protecting aromatic Keteki Joha rice against oxidative damage caused due to Fe^2+^ toxicity.

### 3.5. Differentially Expressed Transcription Factors and Heavy Metal Responsive Genes

Several transcription factors were differentially expressed under Fe^2+^ toxicity (Figure 3C). These include the auxin response factors (ARF), basic helix-loop-helix (bHLH), no apical meristem (NAC), myeloblastosis (MYB), basic leucine zipper (bZIP), WRKY, APETALA2/Ethylene-responsive factor (AP2/ERF), C2H2 zinc-finger domain (C2H2) were prevalent (Figure 3C). Briefly, 100 bHLH, 70 bZIP, 121 NAC, 56 C2H2, 75 MYB, 81 AP2/ERF, and 69 WRKY transcription factors were differentially expressed in roots and leaves (Supplementary Table S4). Similarly, several metal transporters were differentially expressed under Fe^2+^ toxicity (Figure 3B). About 34 heavy metal-associated domains containing heavy metal-associated protein (HMP), 12 multidrug resistance-associated protein (MRP), 7 metal tolerance protein (MTP), 7 NRAMP, and 11 Zrt and Irt-like protein (ZIP) were differentially expressed (Supplementary Table S5).

### 3.6. DEGs Related to Abiotic Stress and Nutrient Deficiency

Some well-known abiotic stress and nutrient deficiency-related genes were also observed among the DEGs. The auxin efflux carrier component genes were highly upregulated in roots, whereas they were either downregulated or unexpressed in leaves (Supplementary Figure S3). Abiotic stress-related genes *9-cis-epoxycarotenoid dioxygenase (OsNCED4/OsNCED3), receptor-like cytoplasmic kinase 253 (OsRLCK253), phosphate dikinase (OsPPDKA)*, and *cyclin-like F-box domain-containing protein (OsMsr9)* were differentially expressed. In addition, submergence tolerance genes *pyruvate decarboxylase (OsPDC4), phosphoenolpyruvate carboxykinase (OsPEPCK), OsARD1/OsSIP2*, and *B12D* protein were differentially expressed. Low nutrient responses genes *haemoglobin 1 (OsHB1), OsHB2*, and *low phosphate root 2 (OsLPR2)* were mostly upregulated (Supplementary Figure S3).

### 3.7. GO and KEGG Pathway Affected by Fe Toxicity

**Figure 4:**
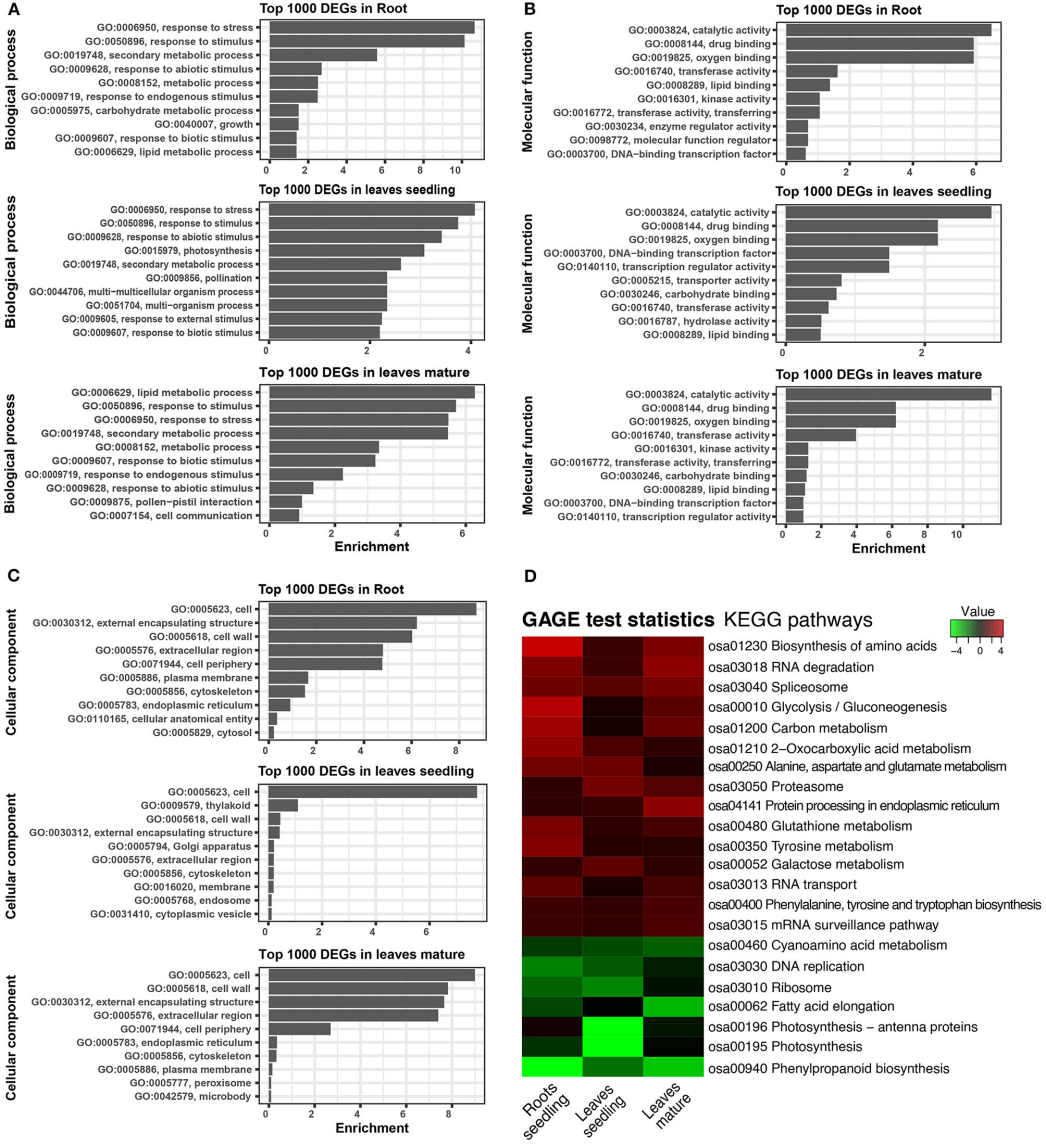
Gene ontology (GO) and KEGG pathway of DEGs. **(A)** Biological process of GO terms associated with top 1,000 DEGs. **(B)** Molecular function GO terms associated with top 1,000 DEGs. **(C)** Cellular component GO terms associated with top 1,000 DEGs. **(D)** Heatmap showing the KEGG pathways of the differentially expressed genes. GAGE statistics were applied, and the top significant pathways were represented using the pathview-web tool.

Gene ontology is helpful for describing the functions of gene products. GO terms of the DEGs in roots, leaves of seedling, and mature stage were very similar. From comparative GO term analysis, top and common biological process GO terms include response to stress (GO:0006950), response to stimulus (GO:0050896), secondary metabolic process (GO:0019748), response to abiotic stimulus (GO:0009628), lipid metabolic process (GO:0006629), etc. (Figure 4A). Similarly, the top and common molecular process GO terms included, catalytic activity (GO:0003824), drug binding (GO:0008144), oxygen binding (GO:0019825), transferase activity (GO:0016740), etc. (Figure 4B). Besides top and common cellular components, GO terms of DEGs also belonged to cell (GO:0005623), external encapsulating structure (GO:0030312), cell wall (GO:0005618), extracellular region (GO:0005576), etc. (Figure 4C).

**Figure 5:**
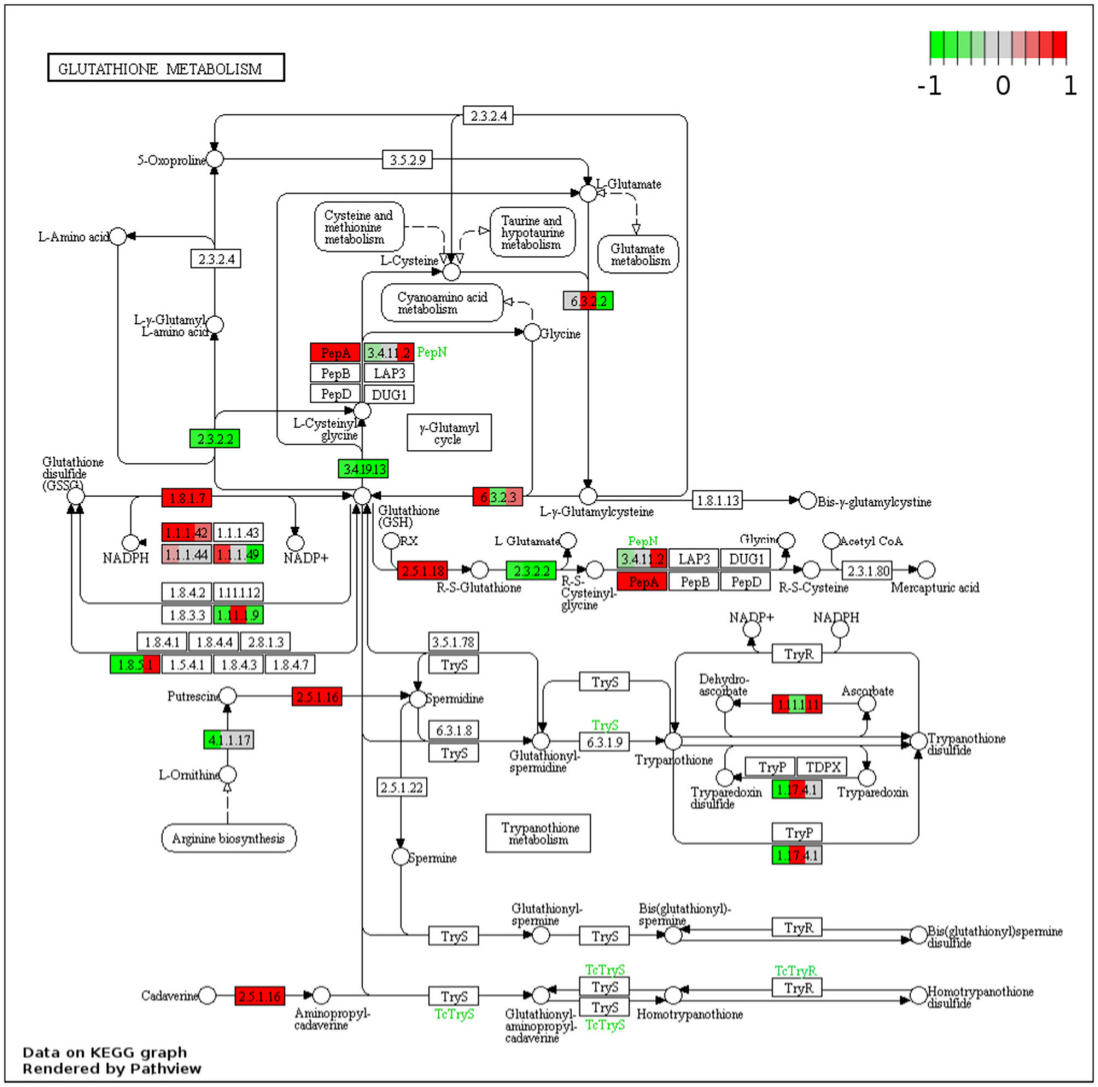
Representative figure showing the multiple states glutathione metabolism pathway induced under Fe^2+^ toxicity. DEGs involved in the pathway are highlighted in red (Upregulated) and green colors (downregulated).

The KEGG pathways of the DEGs were evaluated in the context of adaptation and responses to Fe^2+^ toxicity. Among the several pathways, significantly and differentially expressed pathways were evaluated by the pathview-web tool. Log2FoldChange value of each DEGs was integrated into their respective KEGG gene ID, and their active or passive state was evaluated, and accordingly, multiple states pathways were presented (Figure 4D and Supplementary Figure S4). Top upregulated pathways included biosynthesis of amino acids (osa01230), RNA degradation (osa03018), spliceosome (osa03040), glycolysis/ gluconeogenesis (osa00010), carbon metabolism (osa01200), glutathione metabolism (osa00480) etc (Figures 4D, 5). The most downregulated pathway includes phenylpropanoid biosynthesis (osa00940), photosynthesis (00195), fatty acid elongation (osa00062), ribosome (osa03010), DNA replication (osa03030), etc (Figure 5).

### 3.8. Differential Exon Usage of Genes

**Figure 6:**
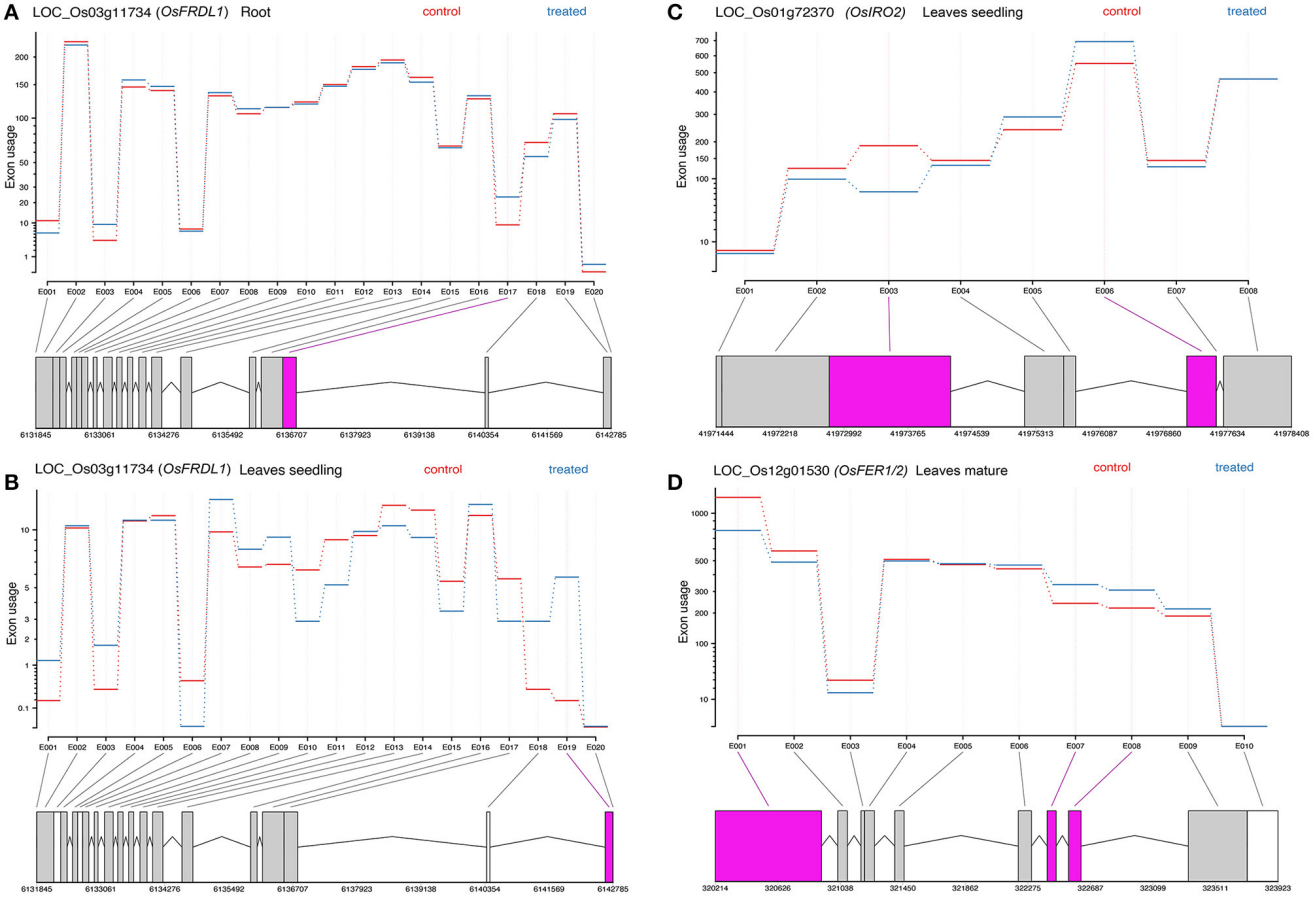
Representative figure showing the differential exon usage of *OsFRDL1, OsIRO2*, and *OsFER1/2* **(A–D)**. Significant differential exons usages are highlighted.

Differential exon usage of the DEGs was inferred in response to Fe^2+^ toxicity. About 173 in root seedling, 692 in leaves seedling, and 1,415 genes in leaves of the mature stage were identified to use different exons (Supplementary Figure S5). Among them, Fe homeostasis genes were also identified to use different exons. In roots, *OsFRDL1, OsNRAMP1, OsVMT*, and *OsTOM3* used different exons between the control and treated groups. Similarly, the *OsFRDL1, OsIRO2*, and *OsMIR* were in leaves seedling, and *OsFER1/2, S-adenosyl-L-methionine synthetase 2 (OsSAM2), OsPEZ2, OsSAM1, OsIDEF2, OsTOM3*, and *OsVMT* used different exons between the control and treated groups (Figure 6).

### 3.9. Validation of Gene Expression

**Figure 7:**
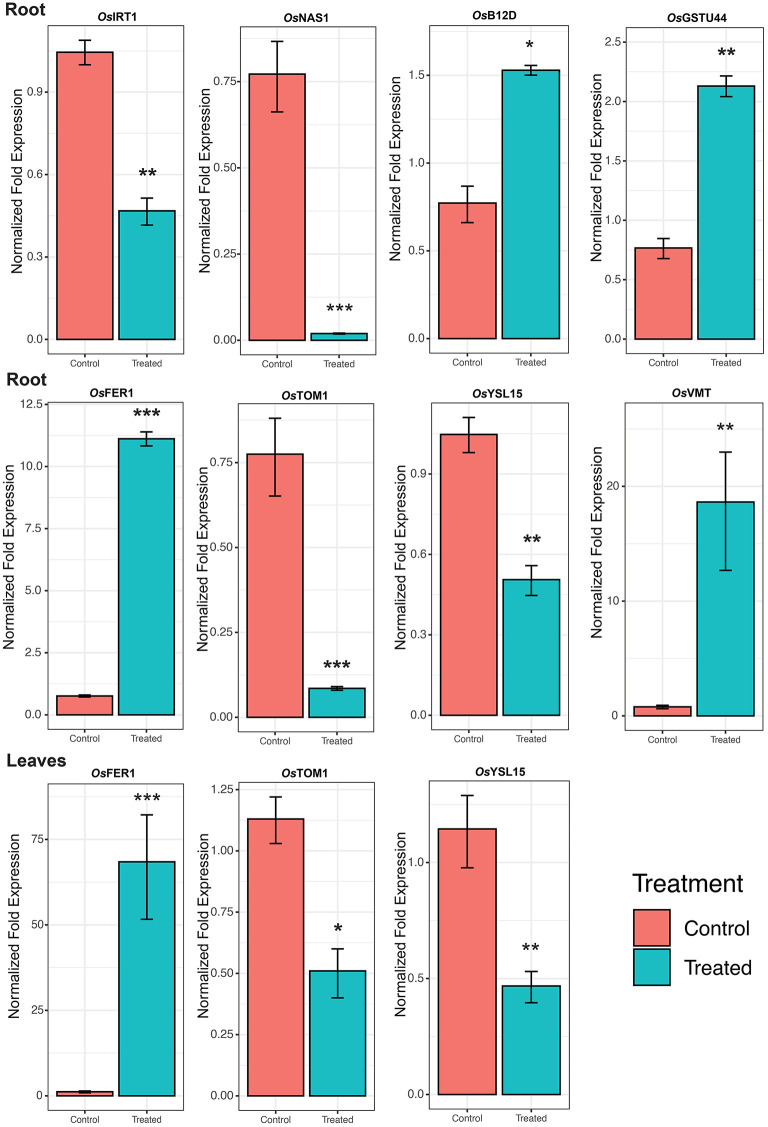
Expression of Fe homeostasis related genes. Skewed error bar represents the results of 3 biological replicates. The significant level indicates the significant difference in means between the control (70 μM of Fe) and treated (2.5 mM Fe) groups estimated by *t*-test in R program. Significant codes with respect to *p*-values are *** 0.001, ** 0.01, and * 0.05.

The expression of RNA-Seq data was validated by qRT-PCR analysis of representative genes (Figure 6). Under Fe^2+^ toxicity, expression of *OsFER1/2* was upregulated in both root and leaves of the seedling stage. The expression of *OsVMT, OsB12D*, and *OsGSTU44* was downregulated in the roots. In contrast, similar to the RNA-Seq results, the *OsIRT1, OsYSL15, OsNAS1*, and *OsTOM1* were downregulated. Similarly, the *OsYSL15* and *OsTOM1* were also downregulated in leaves of the seedling stage (Figure 7).

**Figure 8:**
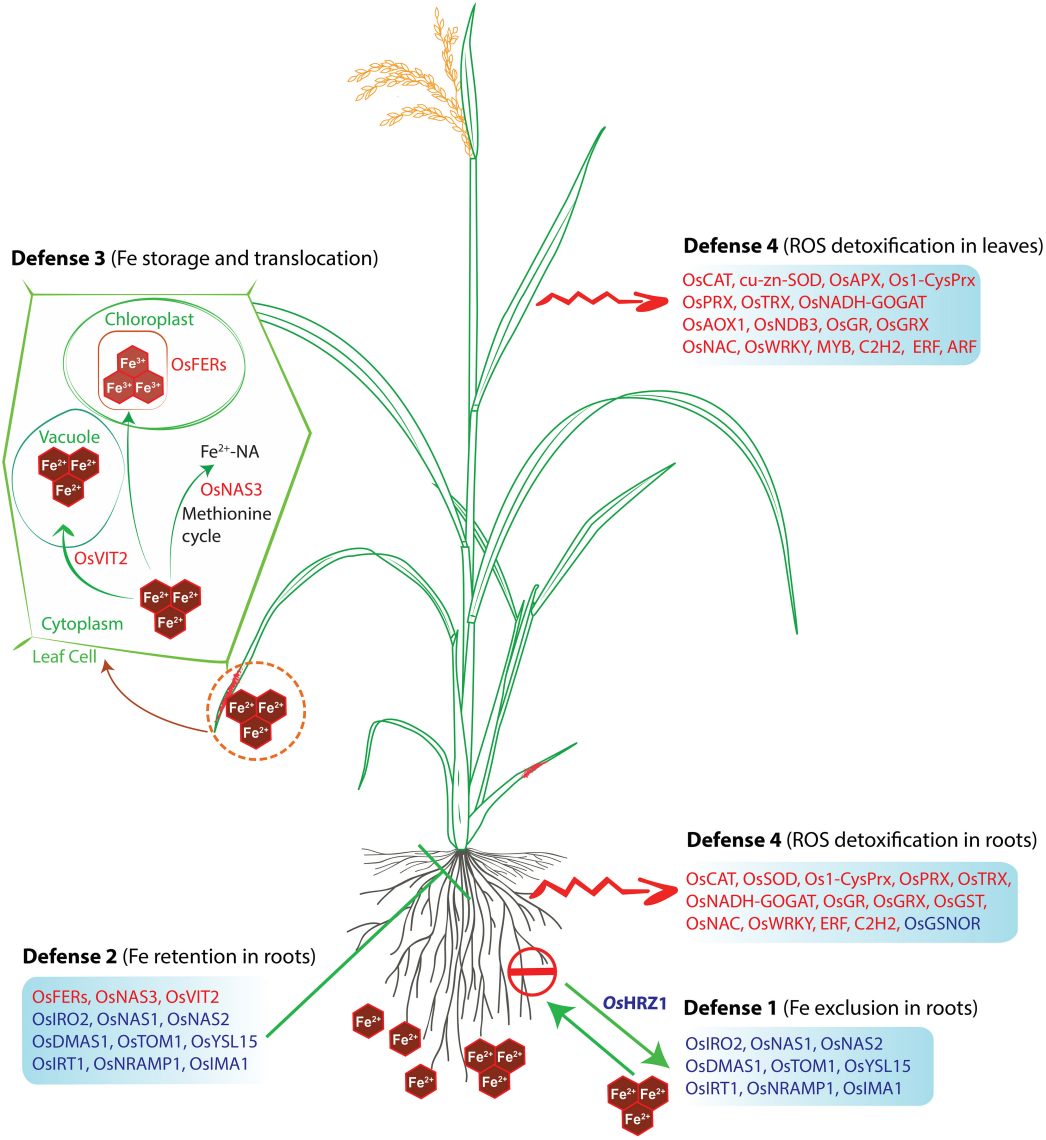
Hypothetical model of the defense mechanisms of rice against Fe toxicity. The model was adapted from Aung and Masuda (2020) and updated with our findings. Red letters indicate upregulated genes, whereas blue letters indicate downregulated genes under Fe-excess. The rice plant used in this model was adopted from the Gramene database and was redrawn.

**Figure 9:**
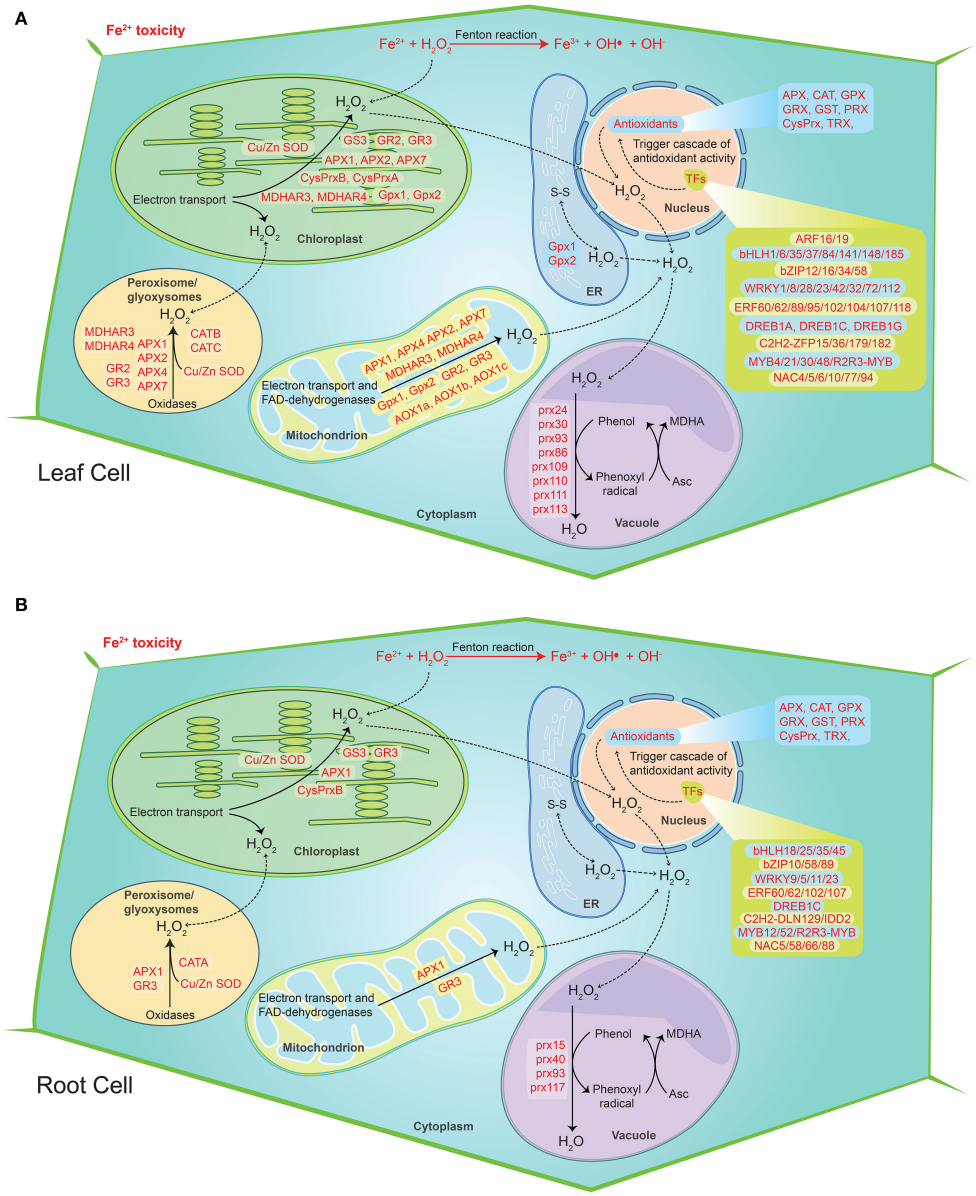
Hypothetical model of the cellular ROS detoxification mechanism under severe Fe^2+^ toxicity in rice. **(A)** ROS detoxification mechanism in root cells. **(B)** ROS detoxification mechanism in leaf cells. The diagram shows the sites of ROS production during the Fe^2+^ toxicity via Fenton reaction. The transcription factors trigger the cascade of antioxidant activity. The genes responsible for H_2_O_2_ removal were highlighted in red color. The solid lines represent the reactions whereas dashed lines represent the transport.

A significant upregulation of ROS Scavenging genes and different abiotic stress-related TFs, genes serve to be the key mechanism involved against severe Fe^2+^ toxicity tolerance. Accordingly, a new defense mechanism is hypothesized that alleviates the excess Fe^2+^ in addition to the other mechanism of defense against Fe^2+^ toxicity (Figures 8, 9).

## 4. Discussion

Fe^2+^ toxicity significantly inhibits the growth and productivity of rice. Fe^2+^ toxicity interrupts the metabolism and functioning of plants, affecting root and shoot development (Aung et al., 2018; Regon et al., 2021). A previous study reported the toxic effect of 2.5 mM Fe^2+^ in aromatic Keteki Joha (Regon et al., 2021). The present study performed a transcriptomic analysis of Keteki Joha under severe Fe^2+^ toxicity (2.5 mM) to investigate how it responds under stress conditions. Fe homeostasis genes were well characterized in Fe deficient condition; however, the molecular mechanism of Fe toxicity in rice plants is still not well empathized (Bashir et al., 2011; Kobayashi et al., 2014). In this study, nearly 69 Fe homeostasis-related genes and their homologs were identified, which were mostly downregulated. The current study shares similarities with the previous microarray result with several other exceptions (Finatto et al., 2015; Aung et al., 2018). Thus, these findings will help to further ameliorate the understanding of Fe homeostasis in rice. Differential exon usages of few Fe homeostasis genes were identified, from which possible alternative splicing of DEGs can be inferred (Anders et al., 2012). However, validation of the predicted genes may be essential to conclude any transcriptional alteration or changes under Fe^2+^ toxicity.

Four different types of tolerance mechanisms have been reported in rice (Aung and Masuda, 2020). However, Fe sensing is crucial at the initial response stage in Fe homeostasis gene expression and regulation based on Fe availability (Kobayashi and Nishizawa, 2014). *OsIDEF1, OsIDEF2*, and *OsIRO2* positively regulate Fe deficiency-inducible genes involved in DMA-based Fe^2+^ acquisition, Fe^2+^ uptake and translocation. In addition, the *OsIDEF1* is directly bound with Fe^2+^ and other divalent metal ions, suggesting an intracellular Fe sensor (Kobayashi, 2019). Moreover, *OsHRZ1* and *OsHRZ2* can also bind with Fe *via* hemerythrin domains, thus characterized as another Fe sensor candidate. Their knockouts have negatively modulated the response to Fe-deficiency in rice. In rice, *OsIDEF1* controls the expression of both *OsHRZ1* and *OsHRZ2* (Li et al., 2019a). However, in this study, the *OsIDEF1* gene was not differentially expressed among all the tissues. *OsHRZ1* being downregulated, suppression of Fe transporter genes at transcriptional level are quite unlike under severe Fe^2+^ conditions. Besides, *OsHORZ1*, a haemerythrin domain-containing protein, was also downregulated, which is known to repress *OsHRZ* functions (Kobayashi and Nishizawa, 2014, 2015; Kobayashi, 2019). In this context, further study of transcription factors in contrasting genotypes may provide a better understanding of their downstream regulation and proper functioning under Fe^2+^ toxicity. However, Fe-excess responses in rice plants are thought to be partially independent of Fe-deficiency (Kobayashi and Nishizawa, 2014). A positive Fe-deficiency regulator *OsPRI1*, a target gene of *OsHRZ1* was also unexpressed in this study. Despite that, *OsNAS3* was upregulated, which is important for Fe detoxification in the root (Aung et al., 2019). In addition, *OsFRDL1* was also upregulated, which is responsible for root-to-shoot Fe translocation and is believed to play a significant role in the distribution of Fe into old leaves. Thus, *OsFRDL1* is crucial for the minimization of Fe^2+^ toxicity in roots. As a result, *OsFER1/OsFER2* and *OsVIT2* were also highly upregulated in leaves, thus defending the Fe overload. The current study is reasonably supported by the proposed Fe-excess defense mechanisms (Aung and Masuda, 2020). In exception, *OsVMT* was highly upregulated in both root and leaves, and it is known to be expressed where Fe and Zn are highly deposited. Knockout of these genes enhances Fe and Zn accumulation in polished rice grains as DMA increases solubilization of Fe and Zn deposited in the node (Che et al., 2019). Considering the function and expression of *OsVMT* under Fe-excess conditions, it is likely to involve in Fe detoxification. Interestingly, the *OsTOM3* DMA-efflux transporter gene belonging to Zinc-induced facilitator (ZIFL subfamily) was upregulated in all the tissues, thereby involved in metal transport. The characterization of the *OsTOM3* gene needs to be further studied for better clarification about its role in Fe^2+^ toxicity. The *OsPEZ1* induced in root tissue plays a significant role in the efficient translocation of *protocatechuic acid (PCA)* and caffeic acid from roots to shoots in rice (Ishimaru et al., 2011). Previously, it was found that expression of *OsMIT* increases under Fe excess, which is in accordance with the current study, essential for the proper growth and development of rice (Bashir et al., 2011). As reported earlier, *OsYSL9* is involved in iron translocation, particularly from endosperm to embryo in developing seeds (Senoura et al., 2017). The YSL family transporters are responsible for transporting metal-phytosiderophores; however, their role is unclear in rice. *OsYSL9* localizes in the plasma membrane and is believed to participate in the transportation of both iron (II)-nicotianamine and iron (III)-deoxymugineic acid into the cell. The expression of *OsYSL10* is like *OsYSL9* and may also participate in a similar mechanism. *OsYSL13* was expressed in roots, and mature leaves participate in the distribution of Fe from older leaves to new leaves (Zhang et al., 2018). Earlier it was found that *OsNRAMP2* was highly induced in shoots and participates in Cadmium accumulation (Zhao et al., 2018). Various studies have already identified the role of *OsNRAMP2* to participate in Fe accumulation which is found to be upregulated in all the tissues. The *OsARD1* is a metal-binding enzyme and is involved in the production of methionine as it binds with Fe^2+^ and catalyzes the formation of 2-keto-4-methylthiobutyrate (KMTB). In the current study, *OsARD1* showed high upregulation in all the tissue types that might enhance rice tolerance under Fe^2+^ toxicity. Overexpression of *OsARD1* also leads to the tolerance of abiotic stresses like submergence, drought, and salinity (Liang et al., 2019).

The discussion above suggests that the Fe exclusion and retention mechanisms in roots are likely to be insufficient under severe Fe^2+^ toxicity. ROS scavenging remains a key mechanism under severe Fe^2+^ toxicity which needs a better understanding (Aung and Masuda, 2020). In this study, most of the antioxidant genes were highly upregulated which clearly indicate the existence of ROS scavenging mechanism. Recently, few ROS scavenging genes, transcriptome factors and transporter genes were highlighted as probable candidates for the ROS scavenging system (Finatto et al., 2015; Wu et al., 2017; Aung et al., 2018; Aung and Masuda, 2020). However, specific ROS scavenging genes and their molecular mechanism are still unknown. The current study on RNA-Seq transcriptome explored the possible ROS scavenging system in rice under severe Fe^2+^ toxicity. Accordingly, a new defense mechanism (defense 4), i.e., ROS detoxification has been hypothesized as a possible mechanism of tolerance against severe Fe^2+^ toxicity. Excess Fe^2+^ is lethal to plants under acidic conditions as they catalyze H_2_O_2_ and produce a highly toxic hydroxyl free radical, termed as Fenton reaction (Winterbourn, 1995). Toxic hydroxyl free radical causes lipid peroxidation and promotes programmed cell death (Becana et al., 1998). Lipid peroxidation and programmed cell death can occur under different environmental stresses. However, Fe-mediated lipid peroxidation and programmed cell death results are quite different from others and are termed Ferroptosis (Distéfano et al., 2020). Rice has developed several Fe tolerance mechanisms, but only the ROS homeostasis was hypothesized to be essential and important in the tolerance mechanism in severe conditions (Aung et al., 2018; Aung and Masuda, 2020). Under Fe^2+^ toxicity, higher production of H_2_O_2_ was reported (Aung et al., 2018; Regon et al., 2021). H_2_O_2_ can be synthesized mostly in every cellular compartment, viz. peroxisomes, chloroplasts, mitochondria, nucleus, vacuoles, etc. CAT was reported to be mainly restricted only in peroxisomes with a very high concentration, thus removing H_2_O_2_ (Smirnoff and Arnaud, 2019). In concordance with previous reports, all three CAT genes were upregulated in roots and leaves of seedling and mature stages. Further, the involvement of these particular CATs was confirmed by KEGG analysis.

Most of the APX, ferredoxin, and thiol-based peroxidase such as PRX genes were upregulated in both roots and leaves, which are important for the removal of H_2_O_2_ in chloroplast using NADPH and photosynthetic electron transport *via* ferredoxin (2Fe-2S, iron-sulfur cluster binding domain-containing protein) as the ultimate reductant (Smirnoff and Arnaud, 2019). Several POX genes were upregulated, which are responsible for scavenging H_2_O_2_ by oxidizing various secondary metabolites. Removal of mitochondrial H_2_O_2_ requires a series of enzymatic detoxification processes. Mn-SOD scavenges superoxide radicals formed *via* mitochondrial electron transport chain (ETC). However, *Mn-OsSOD1* was not found to be differentially expressed in roots, whereas it was fractionally upregulated in leaves of both stages. The remaining H_2_O_2_ is detoxified by the PRX-TRX system or by the enzymes of the ascorbate-glutathione cycle (Smirnoff and Arnaud, 2019). PRX-TRX plays an important role in detoxifying H_2_O_2_ in plants (Wang et al., 2016). The *Os1-CysPrxB* is highly expressed across all tissue types. In addition, upregulation of *OsTRX24, OsTRX23, OsTRX2* etc. denotes the PRX-TRX regulation for detoxification of H_2_O_2_ in mitochondria under Fe^2+^ toxicity.

Among the different antioxidants, GSTs were one of the most differentially expressed genes under Fe^2+^ toxicity. Most upregulated GSTs are of the tau class, which are well-known for heavy metal detoxification due to the high affinity of metals to its thiol(-SH) group and as a precursor of phytochelatins (PCs) (Jagodzik et al., 2018). In this study, the tau GSTs such as *OsGSTU6, OsGSTU8, OsGSTU17, OsGSTU19, OsGSTU30, OsGSTU44*, etc., were highly upregulated in both root and leaves and thus expected to play an essential role in Fe^2+^ mediated cellular detoxification. The *OsGSTU17* possesses diverse regulatory mechanisms in response to abiotic stresses, whereas overexpression of *OsGSTU30* was reported to promote tolerance to Chromium (Cr) and drought stress (Yang et al., 2009; Srivastava et al., 2018). In addition, KEGG analysis revealed the glutathione pathways as one of the most actively regulated pathway in this study. Glutathione synthase *viz*. *OsNADH-GOGAT1* and *OsNADH-GOGAT2* were identified as highly upregulated genes in roots involved in the glutathione synthesis pathway, whereas *OsNADH-GOGAT1* was identified in leaves. Moreover, alternative respiratory pathway component genes, like alternative oxidase (AOX) and NAD(P)H dehydrogenase (NDs), play an essential role in minimizing ROS production (Vanderauwera et al., 2005; Rhoads et al., 2006; Keunen et al., 2015; Huang et al., 2016). *OsAOX1a, OsAOX1b, OsAOX1c*, and *OsNDB3* were highly upregulated in leaves, thus suggesting the existence of an alternative respiratory pathway under Fe^2+^ toxicity. As described previously, the modulation of this mechanism was observed under cold stress in rice (Ito et al., 1997). Overall, expression of GSH, APX, MDHAR, DHAR, and GR indicates the active role of the ascorbate-glutathione cycle under Fe^2+^ detoxification which has been also affirmed by the KEGG pathway analysis.

Iron homeostasis is a complex network that is regulated by various transcription factors. The tale of transcription factors involved in the regulation of different Fe-acquisition was well characterized in *Arabidopsis*, whereas it is comparatively less explored in rice. In *Arabidopsis*, 16 bHLH transcription factors were described to be involved in Fe deficiency responses (Gao et al., 2019). In this study, several other bHLH TFs were differentially expressed, which need to be further elucidated. Moreover, in *Arabidopsis*, MYB, WRKY, AP2/ERF, and C2H2 TFs were involved in Fe acquisition, translocation, inhibition, and modulation of other Fe homeostasis genes (Gao et al., 2019). In rice, *OsIDEF1* (ABI3/VP1 TF family), *OsIDEF2* (NAC TF family), and *OsARF16* (ARF family) reported to modulate the expression of Fe-related genes and integrate auxin signals, respectively, thus play a critical role in Fe homeostasis (Ogo et al., 2008; Shen et al., 2015; Gao et al., 2019). Apart from the above TFs, several other TFs were differentially expressed under Fe^2+^ toxicity, which might involve Fe homeostasis regulation in rice.

Furthermore, TFs such as bHLH, bZIP, ERF, WRKY, NAC, and MYB were important for the protection of ROS mediated oxidative damage by triggering ROS scavenging related genes in plants (Mittler et al., 2004; Yu et al., 2017; He et al., 2018). However, the knowledge of TFs under Fe^2+^ toxicity is limited. Recently, NAC (*OsNAC4, OsNAC5, OsNAC6*) and WRKY TFs were hypothesized to regulate severe Fe^2+^ toxicity tolerance in rice (Aung and Masuda, 2020). Besides, *GSNOR* (downregulated in this study) was reported to promote root tolerance to Fe toxicity *via* a nitric oxide pathway (Li et al., 2019b). WRKY transcription factors are well-known to play a key role in both biotic and abiotic stress tolerance as well as plant hormones signal transduction and the MAPK signaling cascade in plants (Liu et al., 2007; Adachi et al., 2015; Kim et al., 2016; Jiang et al., 2017). Among the several upregulated WRKY TFs, OsWRKY8 and OsWRKY71 were involved in various biotic and abiotic stresses in rice (Liu et al., 2007; Kim et al., 2016). Moreover, APETALA2/Ethylene Response Factors (AP2/ERF) are well-known to regulate numerous abiotic stresses (Müller and Munné-Bosch, 2015; Phukan et al., 2017). Several AP2/ERF transcription factors were upregulated under Fe^2+^ toxicity. Interestingly, dehydration-responsive element-binding proteins (DREBs) transcription factors were downregulated in roots, whereas upregulated in leaves. Overall, differentially expressed TFs under Fe^2+^ toxicity are very intense, which need to be further studied to elucidate their specific role in understanding Fe^2+^ toxicity in rice. However, identification of their downstream target genes will remain important to uncover the possible signaling pathways in rice. Recent computational analysis has identified several *cis*-regulatory elements (CREs) and conserved motifs among the Fe homeostasis related genes which are important for their regulation by transcription factors (Kakei et al., 2021). Fe storage-related genes were reported to simultaneously possess the conserved downstream core element 1 (DCEp1) and Fe deficiency-associated motif 1 (FAM1) motifs, whereas Fe uptake genes tend to possess the FAM1, DCEp1, and TATABOX5 motifs (Kakei et al., 2021). Similar computational and molecular approaches of this study may be helpful in identifying novel mechanisms associated with Fe^2+^ toxicity tolerance in rice.

Comparative KEGG analysis revealed that genes involved in the Mitogen-activated protein kinases (MAPK) signaling pathway are highly upregulated in leaves, under Fe^2+^ toxicity. MAPK signaling pathway is a common defense response of plants for various abiotic stresses (Zhang and Klessig, 2001; Liu and He, 2017). Along with several MPK genes, *OsMPK3* was upregulated in leaves, which are known as a stress tolerance gene (Jagodzik et al., 2018). In addition, *pathogen-related protein 1 (OsPRI1)* was also upregulated in all tissue types, which plays an essential role in the plant metabolism in response to biotic and abiotic stresses (Muhammad et al., 2019).

In addition, upregulation of low nutrient responsive genes indicates nutrient deficiency upon Fe^2+^ toxicity. Apart from the ROS homeostasis genes, differential expression of various abiotic stress-related genes might indicate the existence of multiple stress tolerance strategies in rice. Besides all the above, *L-lactate dehydrogenase B (LDH-B)* was the topmost upregulated gene in roots, which catalyzes the reversible NAD-dependent interconversion of pyruvate to L-lactate. KEGG pathway analysis revealed that it is involved in glycolysis/gluconeogenesis, cysteine and methionine metabolism, pyruvate metabolism, propanoate metabolism, and biosynthesis of secondary metabolites. Its stereoisomer *D-2-hydroxyglutarate dehydrogenase/D-lactate dehydrogenase* was recently characterized to be conferred multiple abiotic stresses by maintaining cellular homeostasis in rice (Mitsuhara et al., 2008). Thus, further study about LDH-B may be helpful to understand its function under Fe^2+^ toxicity in rice.

## Conclusion

In this study, ROS homeostasis was identified as the key mechanism for Fe^2+^ detoxification under severe Fe^2+^ toxicity, regulated by transcription factors. Future molecular research work targeting the characterization of the strongly induced genes will be important to understand the overall Fe^2+^ detoxification mechanism in rice. In addition, the genes identified in this study may serve as a valuable insight for further research and development of rice genotypes for Fe^2+^ toxicity tolerance.

## Data Availability Statement

The original contributions presented in the study are publicly available. This data can be found here: Bioproject: PRJNA680441. BioSamples: SAMN16879912, SAMN16879913, SAMN16879914, SAMN16879915, SAMN16879916, and SAMN168799.

## Author Contributions

PR: conceptualization, methodology, writing–original draft preparation, data curation, and analysis. SD: manuscript writing and analysis. MR: methodology. AP: manuscript writing. UC: editing. BT: investigation, writing–reviewing, and editing. AT: reviewing and supervision. SP: reviewing, editing, and supervision. All authors contributed to the article and approved the submitted version.

## Funding

The RNA Sequencing was funded by the Department of Biotechnology, Government of India with a grant no. DBT-NER/AGRI/29/2015.

## Conflict of Interest

The authors declare that the research was conducted in the absence of any commercial or financial relationships that could be construed as a potential conflict of interest.

## Publisher's Note

All claims expressed in this article are solely those of the authors and do not necessarily represent those of their affiliated organizations, or those of the publisher, the editors and the reviewers. Any product that may be evaluated in this article, or claim that may be made by its manufacturer, is not guaranteed or endorsed by the publisher.

## Supplementary Material

Supplementary Figure S1 Representative figure showing the morphology of Keteki Joha grown under control and Fe Excess (2.5 mM) conditions.

Supplementary Figure S2 Venn diagram showing the top 500 Up and downregulated common DEGs among the tissue samples. The venn diagram were created by using the venn diagram software of VIB/UGent Bioinformatics & Evolutionary Genomics.

Supplementary Figure S3 Venn diagram showing the common Fe homeostasis DEGs among the tissue samples. The venn diagram were created by using the venn diagram software of VIB/UGent Bioinformatics & Evolutionary Genomics.

Supplementary Figure S4 Gene ontology showing the Molecular function terms associated with top 1000 differentially expressed genes. The top 10 biological process terms were represented by applying Fishers exact statistical test and classic topGO package algorithm in R program. The ggplot2 package of R program was used to create the graphical representation.

Supplementary Figure S5 Gene ontology showing the Cellular component terms associated with top 1000 differentially expressed genes. The top 10 biological process terms were represented by applying Fishers exact statistical test and classic topGO package algorithm in R program. The ggplot2 package of R program was used to create the graphical representation.

Supplementary Figure S6 Most significant KEGG pathways under Fe^2+^ toxicity. Multiple states pathway was rendered by Pathview-web tool and the DEGs involved in the pathway are highlighted in red (Upregulated) and green color (down-regulated).

Supplementary Figure S7 Venn diagram showing the number of common differentially exon usage DEGs in different tissues under Fe^2+^ toxicity.

Supplementary Table S1 RNA-Seq samples showing the number of reads and percentage of uniquely mapped reads into the rice genome.

Supplementary Table S2 Total differentially expressed genes and their annotation in all tissue samples.

Supplementary Table S3 Differentially expressed genes for ROS production and scavenging related genes.

Supplementary Table S4 Differential expression of transcription factors.

Supplementary Table S5 Differential expression of heavy metal related genes.
