# Supplementary material for: Transcriptomic Analysis Revealed Reactive Oxygen Species Scavenging Mechanisms Associated With Ferrous Iron Toxicity in Aromatic Keteki Joha Rice
Source: Front Plant Sci. 2022 Feb 24;13:798580. doi: 10.3389/fpls.2022.798580 (PMC8913046; doi:10.3389/fpls.2022.798580)
Supplement: Supplementary Figure S1 — Representative figure showing the morphology of Keteki Joha grown under control and Fe Excess (2.5 mM) conditions. [file Data_Sheet_1.DOCX]

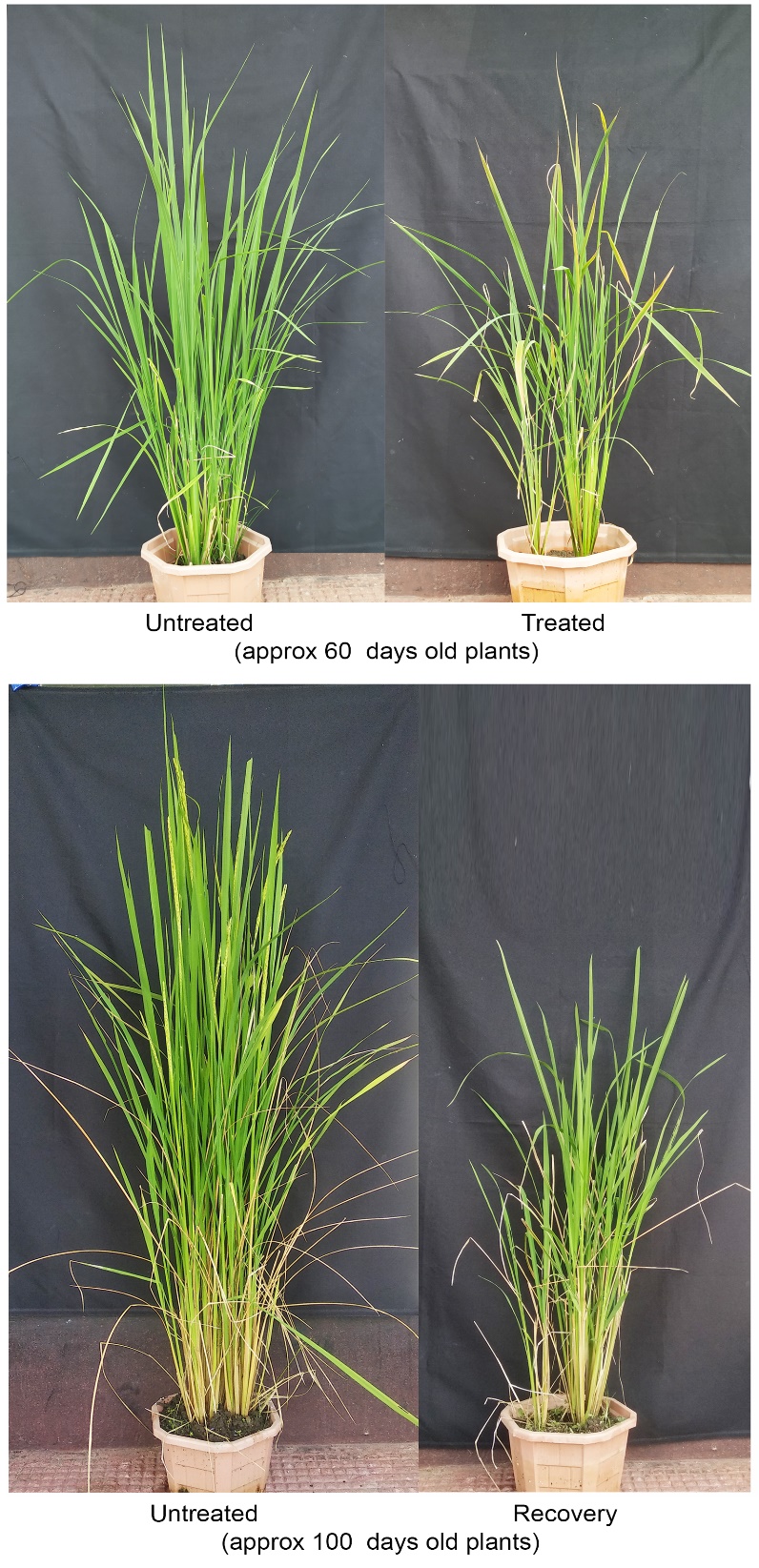


Fig S1: Representative figure showing the morphology of Keteki Joha grown under control and Fe Excess (2.5 mM) conditions.


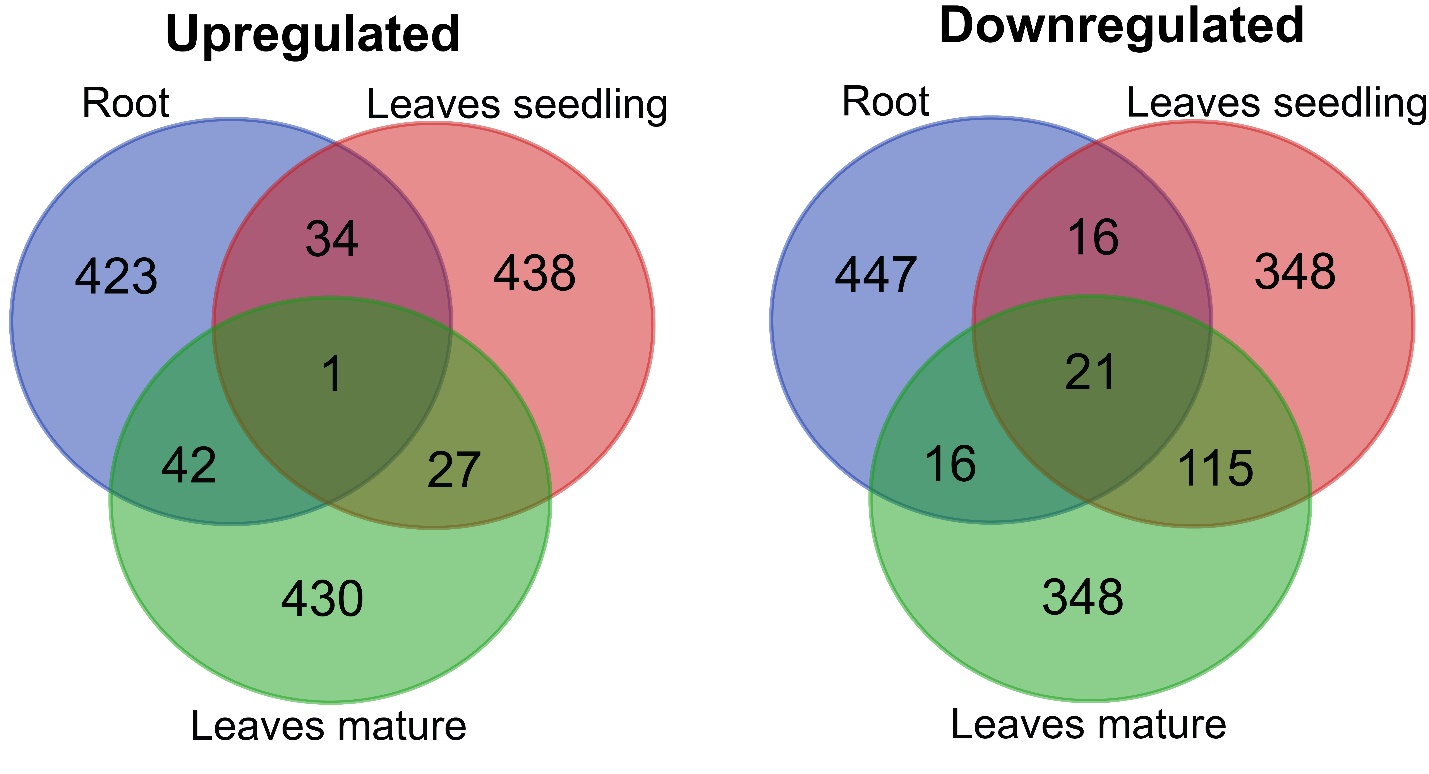


Figure S2: Venn diagram showing the top 500 Up and downregulated common DEGs among the tissue samples. The venn diagram were created by using the venn diagram software of VIB/UGent Bioinformatics & Evolutionary Genomics.


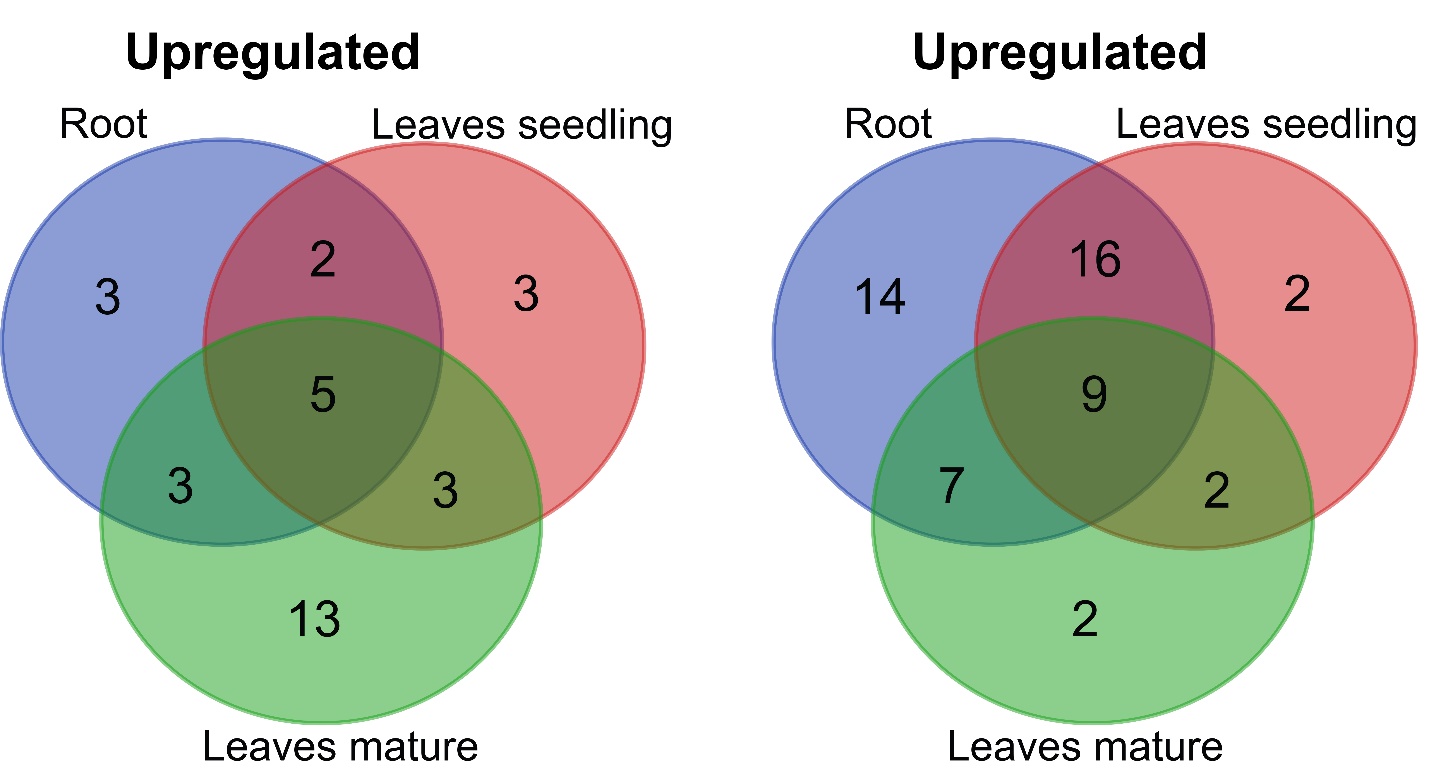


Figure S3: Venn diagram showing the common Fe homeostasis DEGs among the tissue samples. The venn diagram were created by using the venn diagram software of VIB/UGent Bioinformatics & Evolutionary Genomics.


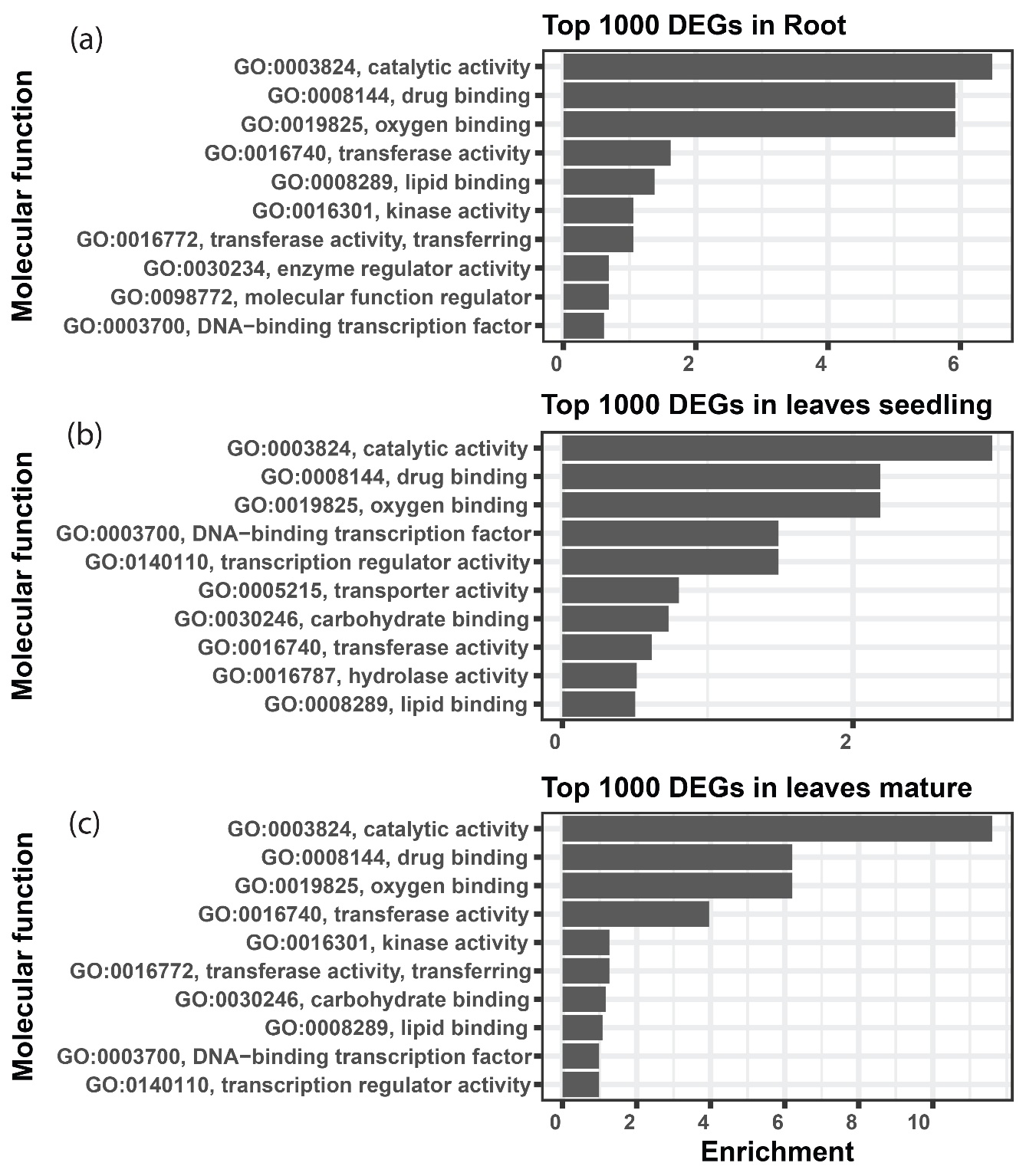


Figure S4: Gene ontology showing the Molecular function terms associated with top 1000 differentially expressed genes. The top 10 biological process terms were represented by applying Fishers exact statistical test and classic topGO package algorithm in R program. The ggplot2 package of R program was used to create the graphical representation.


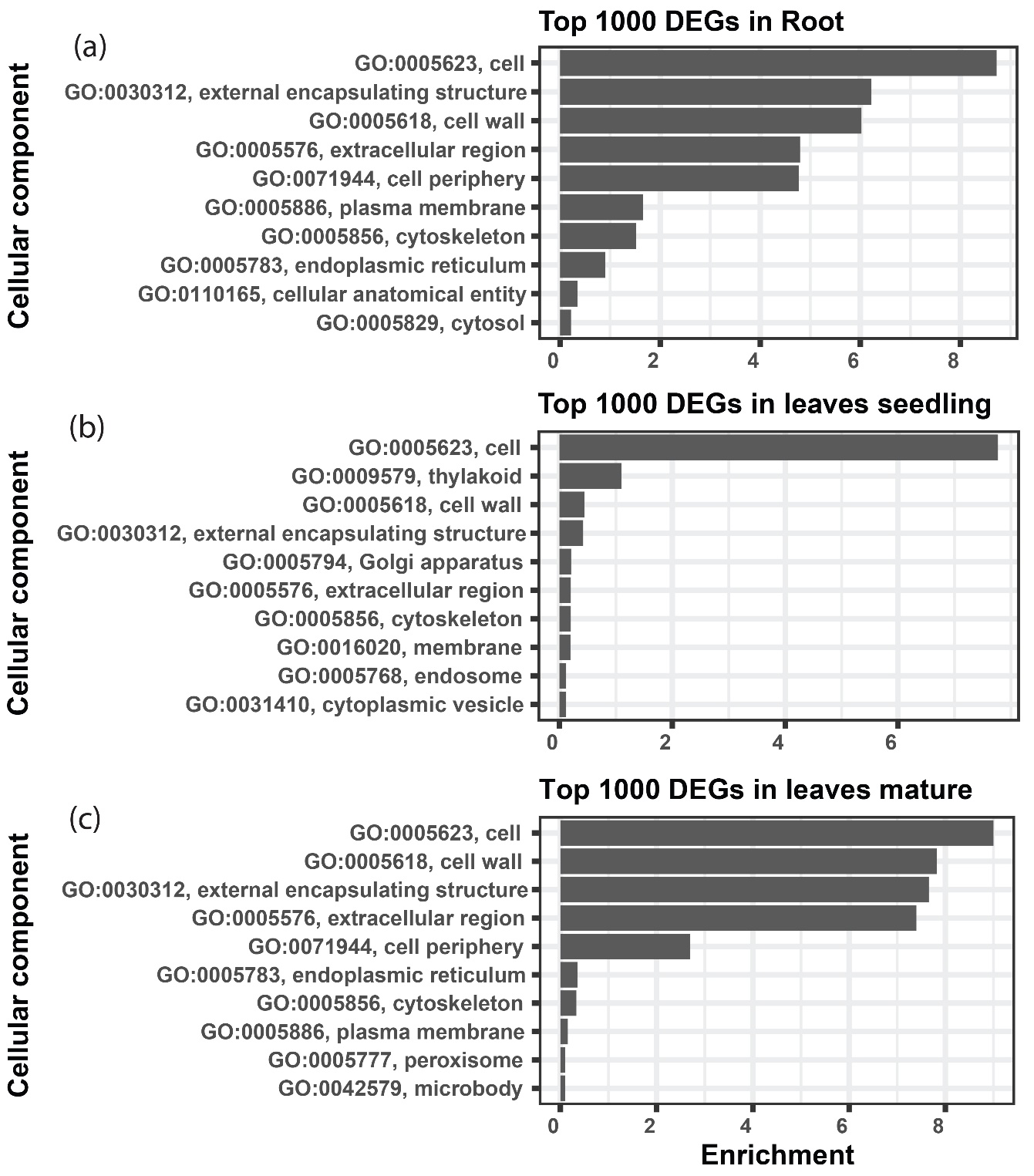


Figure S5: Gene ontology showing the Cellular component terms associated with top 1000 differentially expressed genes. The top 10 biological process terms were represented by applying Fishers exact statistical test and classic topGO package algorithm in R program. The ggplot2 package of R program was used to create the graphical representation.

Figure S6: Most significant KEGG pathways under Fe^2+^ toxicity. Multiple states pathway was rendered by Pathview-web tool and the DEGs involved in the pathway are highlighted in red (Upregulated) and green color (down-regulated).


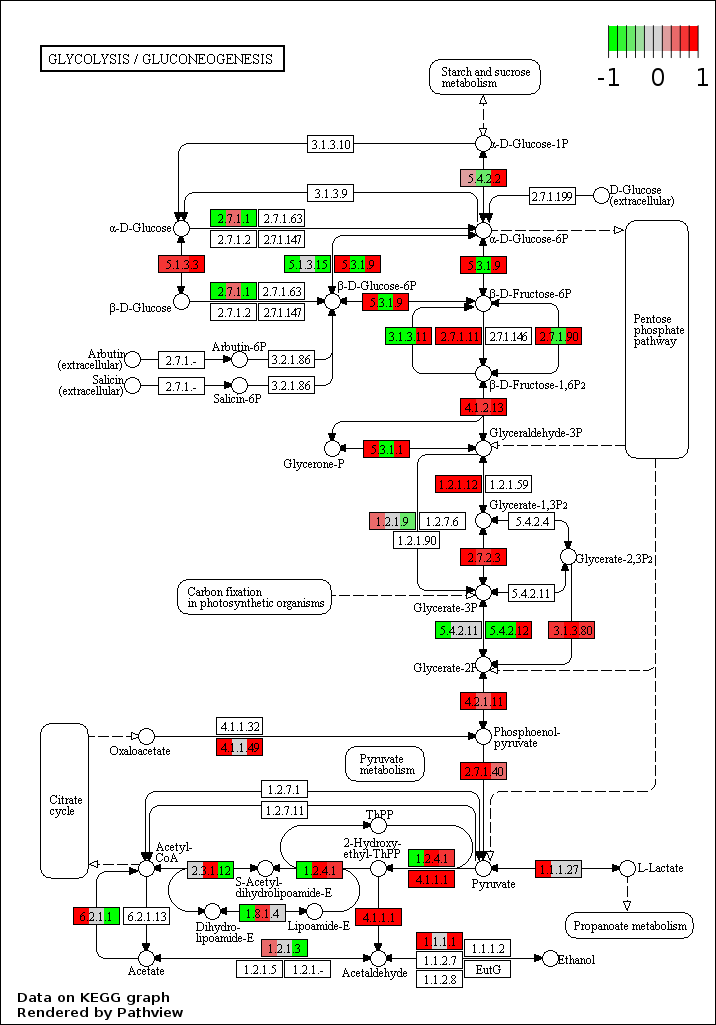


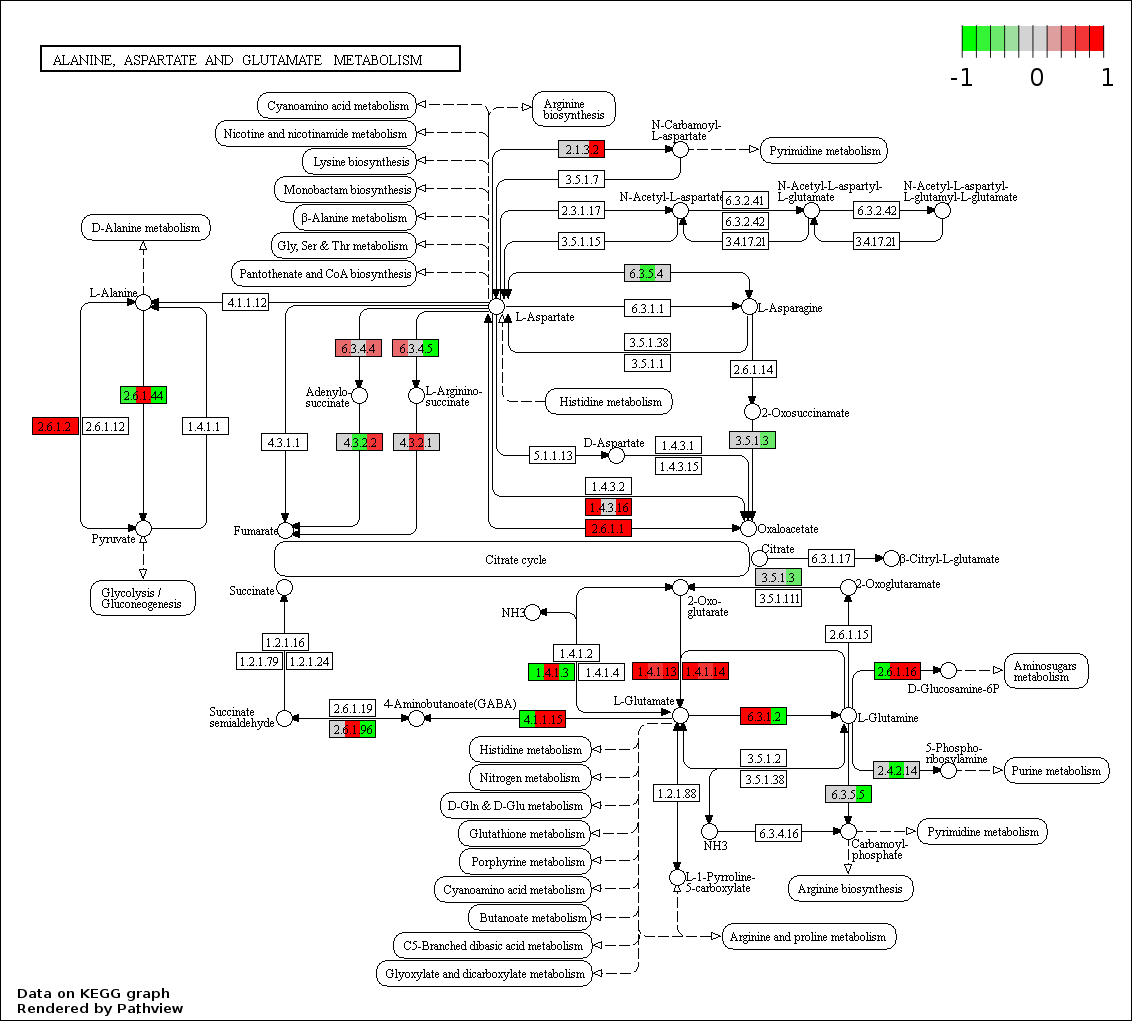


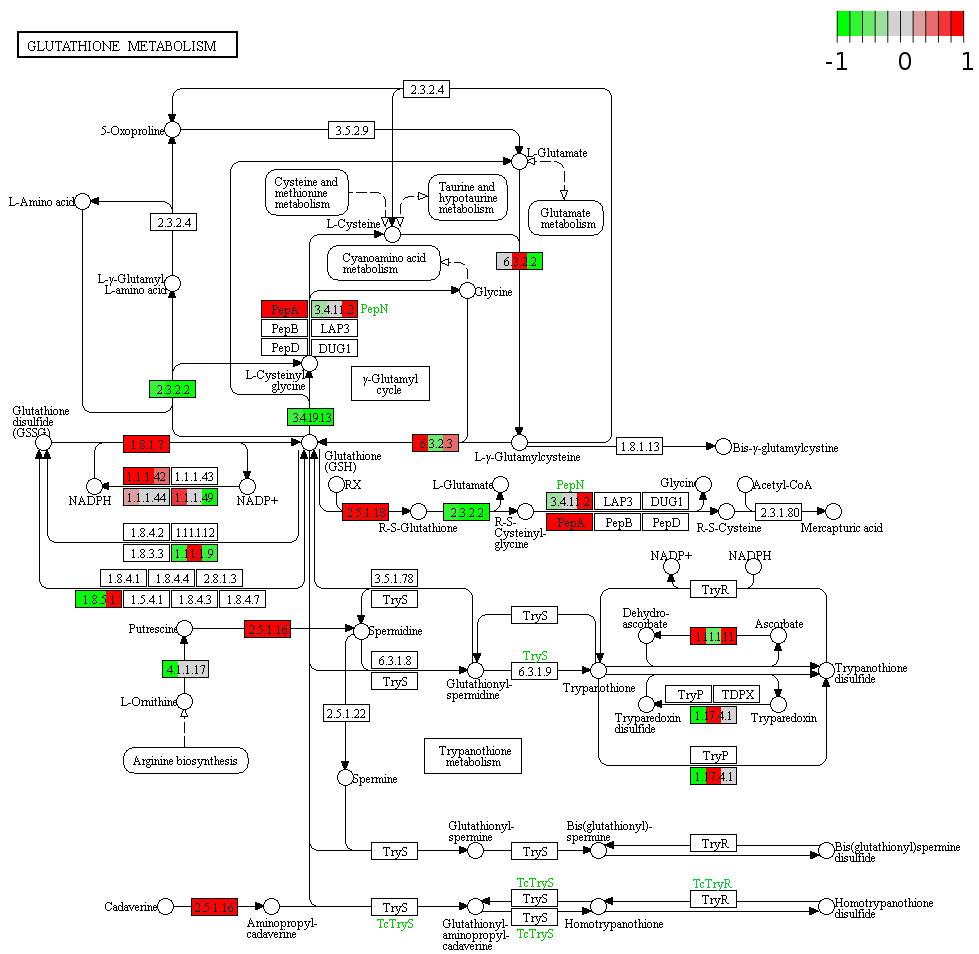


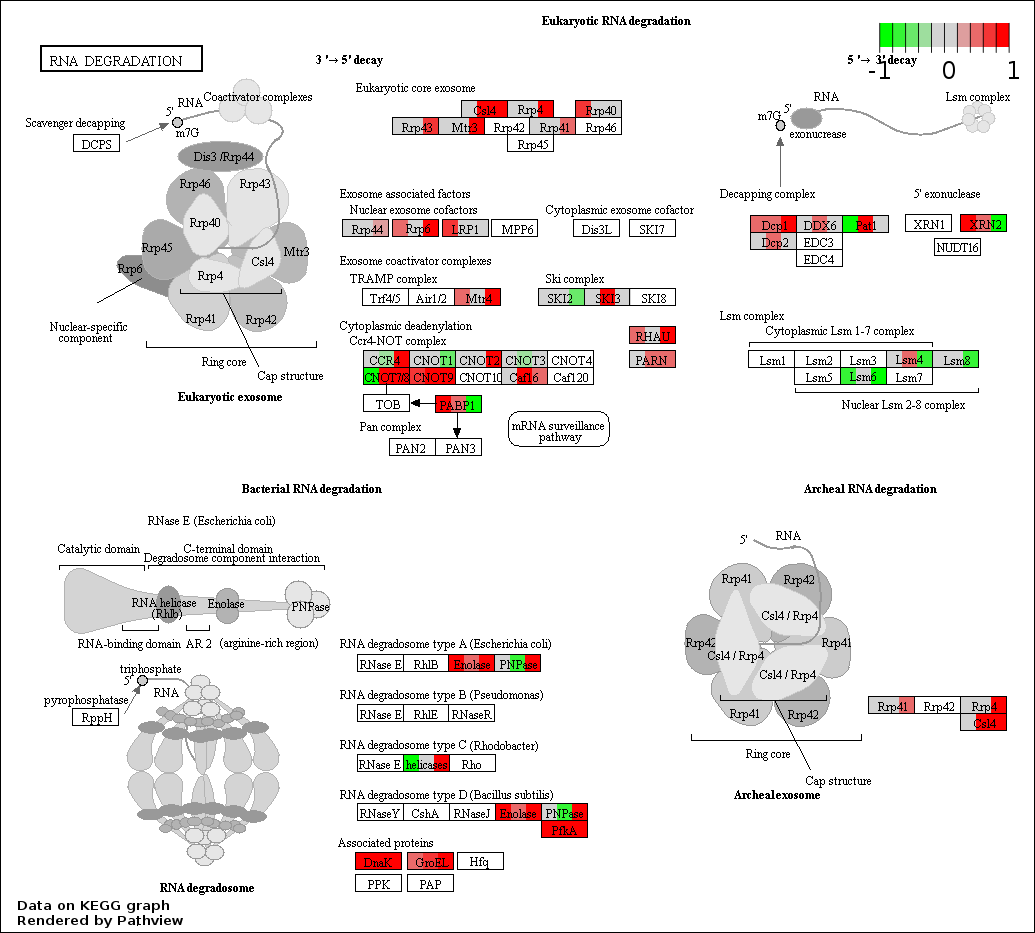


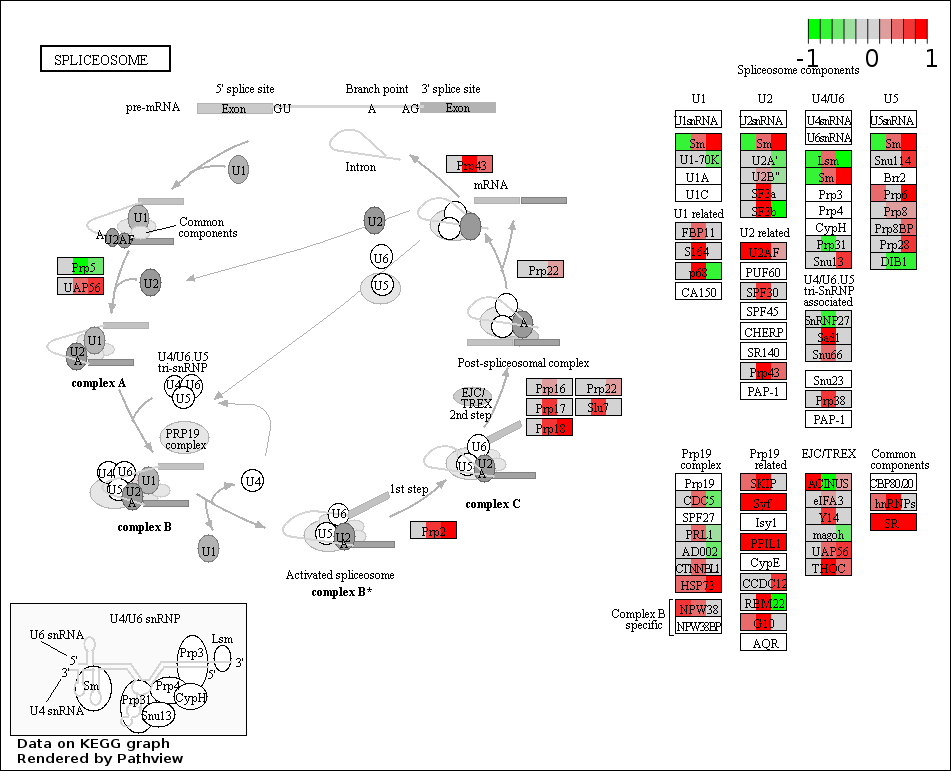


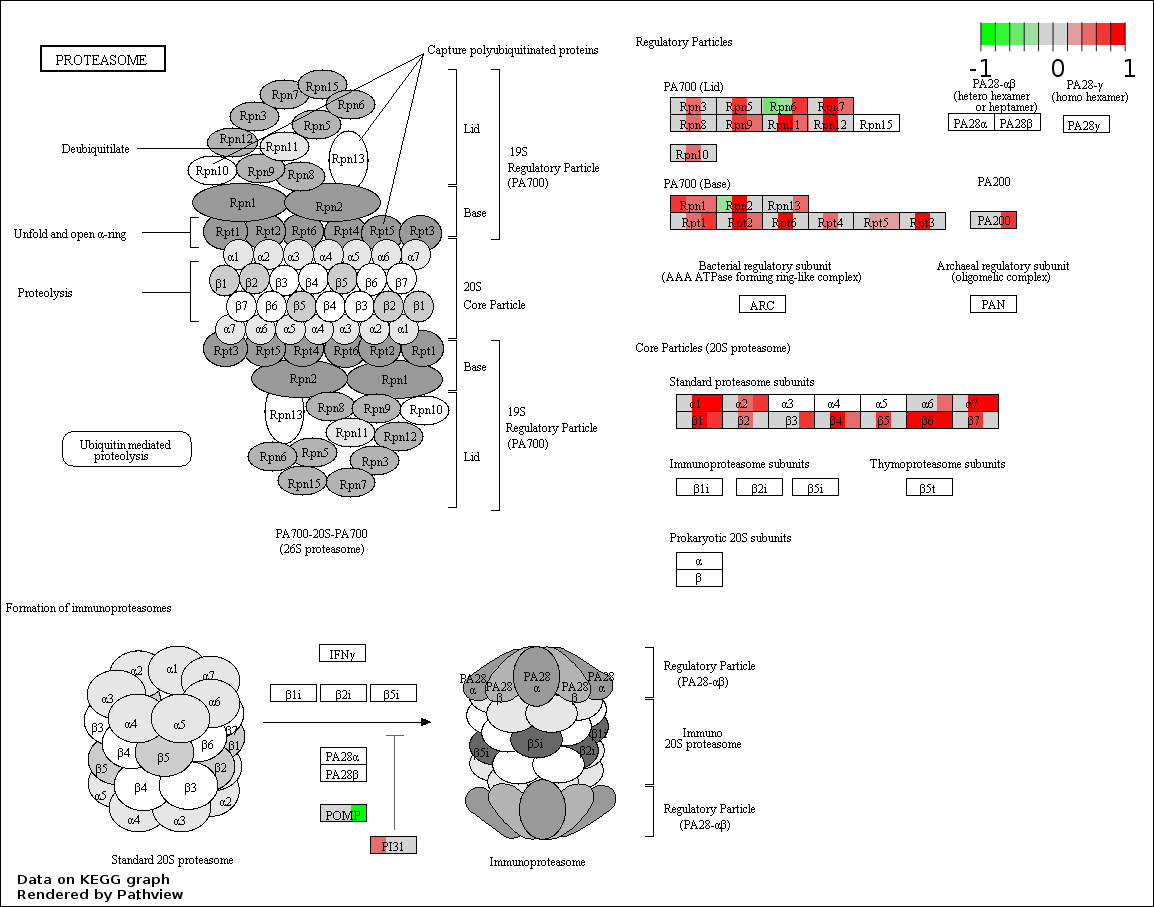


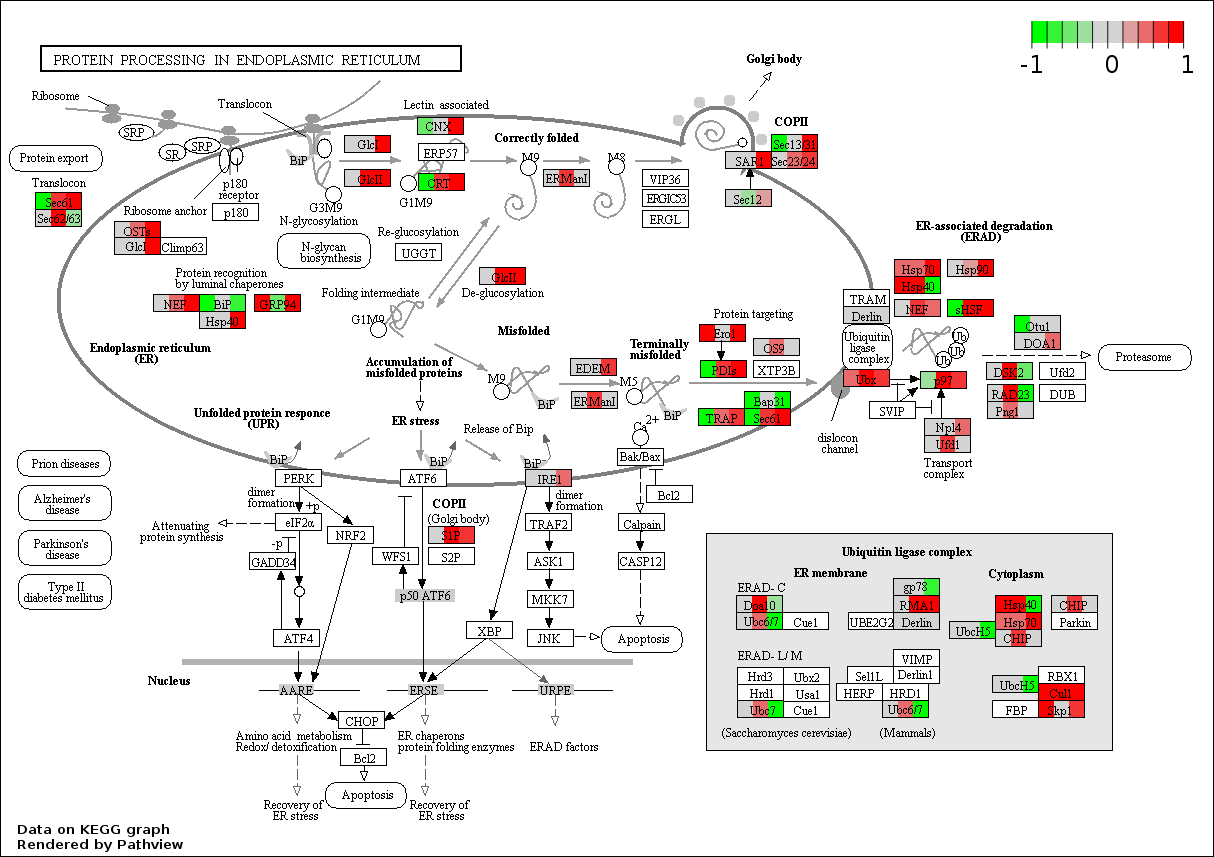


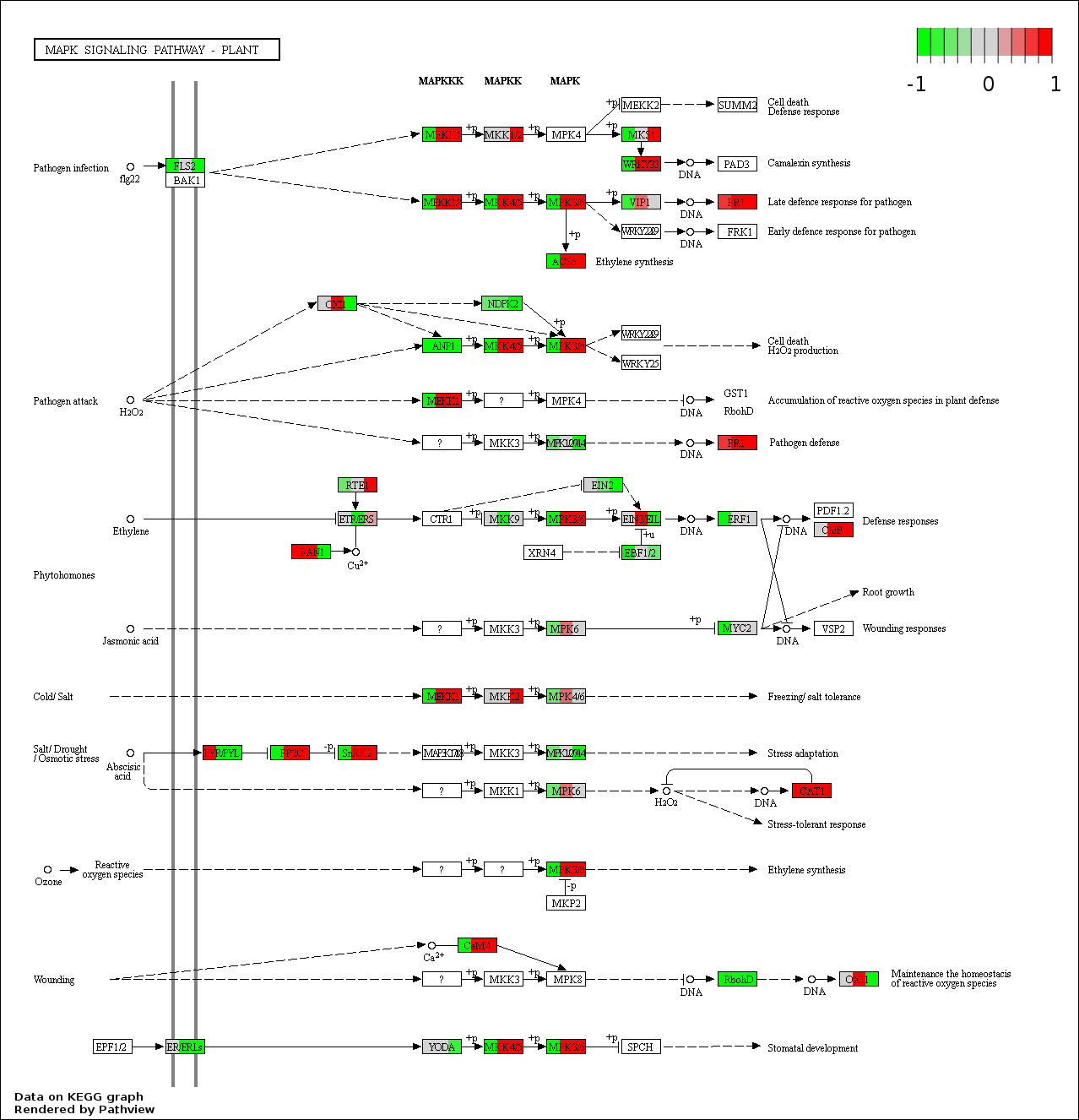


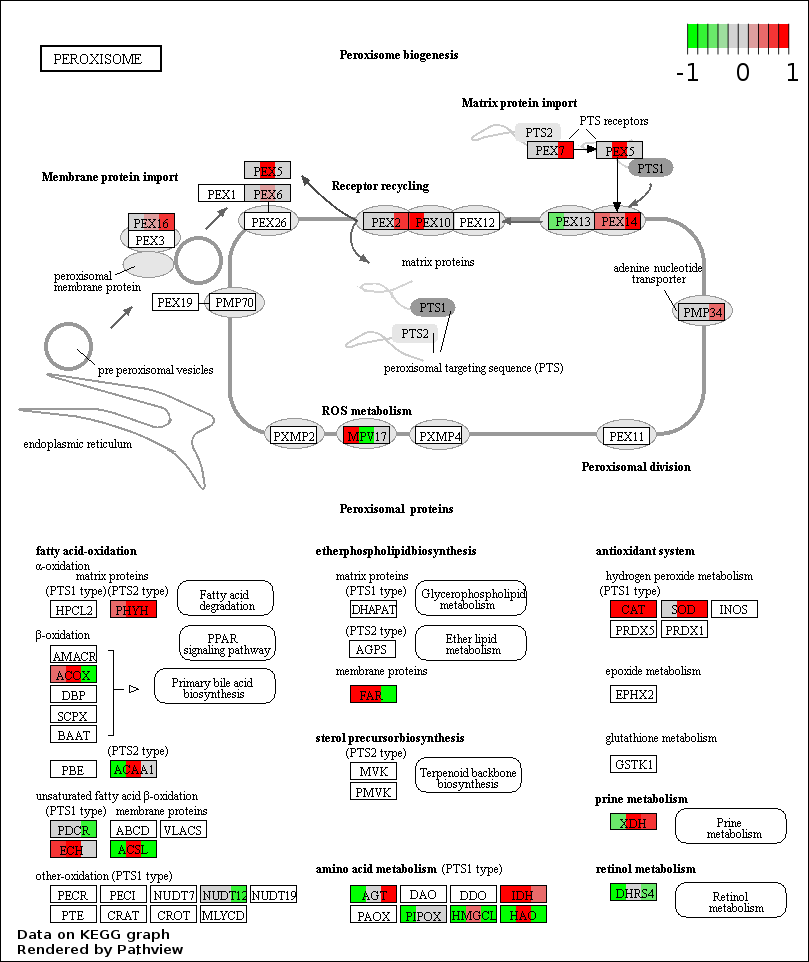


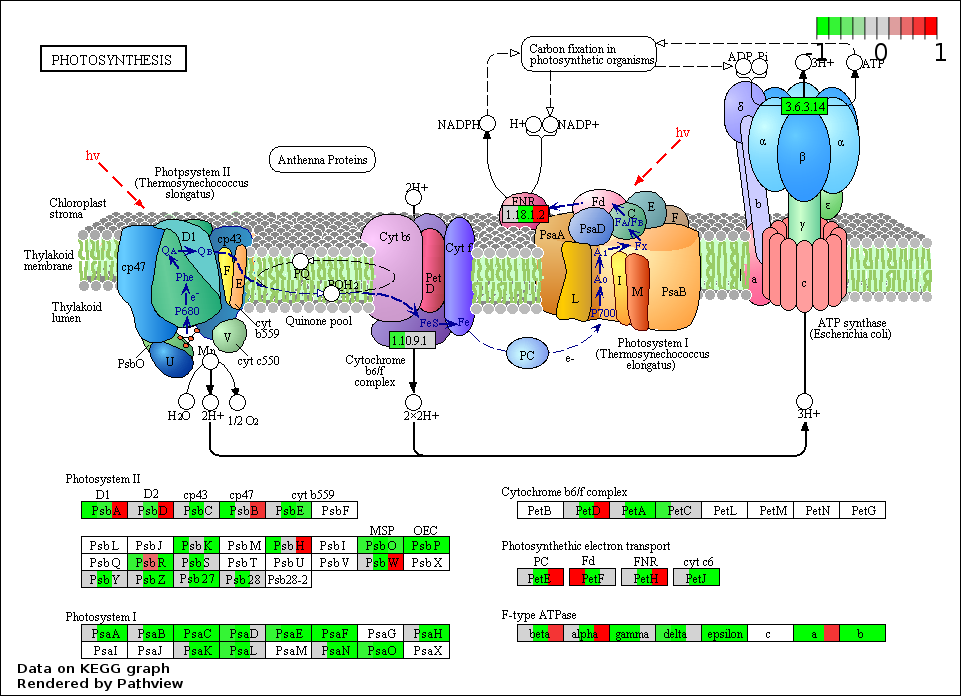


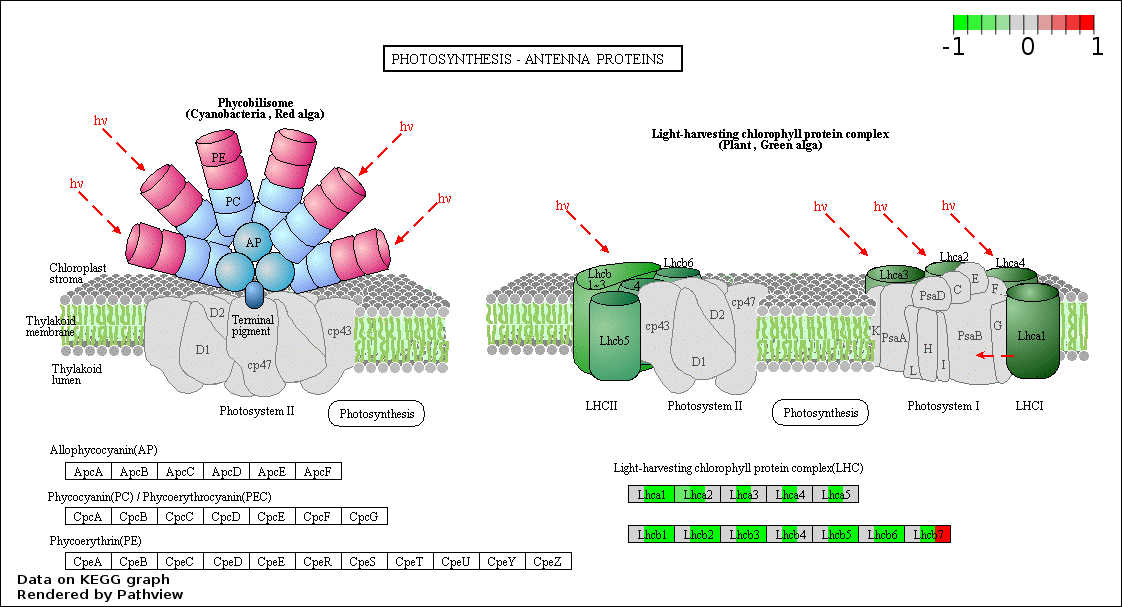


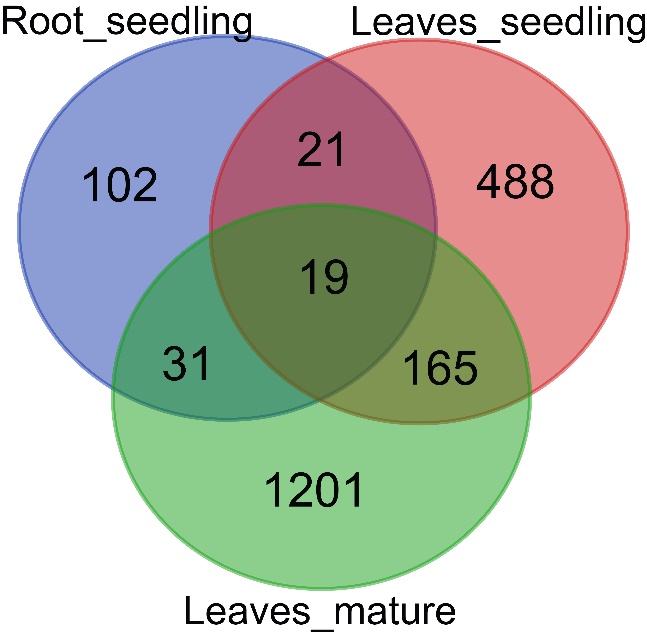


Figure S7: Venn diagram showing the number of common differentially exon usage DEGs in different tissues under Fe^2+^ toxicity.
